# First data of Neandertal bird and carnivore exploitation in the Cantabrian Region (Axlor; Barandiaran excavations; Dima, Biscay, Northern Iberian Peninsula)

**DOI:** 10.1038/s41598-018-28377-y

**Published:** 2018-07-12

**Authors:** Asier Gómez-Olivencia, Nohemi Sala, Carmen Núñez-Lahuerta, Alfred Sanchis, Mikel Arlegi, Joseba Rios-Garaizar

**Affiliations:** 1 Department Estratigrafía y Paleontología, Facultad de Ciencia y Tecnología, Universidad del País Vasco-Euskal Herriko Unibertsitatea (UPV/EHU), Barrio Sarriena s/n, 48940 Leioa, Spain; 2 IKERBASQUE. Basque Foundation for Science, 48013 Bilbao, Spain; 3 Centro UCM-ISCIII de Investigación sobre Evolución y Comportamiento Humanos, Avda. Monforte de Lemos 5 (Pabellón 14), 28029 Madrid, Spain; 4 Aragosaurus-IUCA, Departamento de Ciencias de la Tierra, Facultad de Ciencias, Universidad de Zaragoza, C/Pedro Cerbuna, 12, 50009 Zaragoza, Spain; 5 Museu de Prehistòria de València, Servei d’Investigació Prehistòrica, Diputació de València, Corona 36, València, Spain; 6 Université de Bordeaux, PACEA UMR 5199, Bâtiment B8, Allée Geoffroy Saint-Hilaire, 33615 Pessac, France; 7 Archaeology Program, Centro Nacional de Investigación sobre la Evolución Humana (CENIEH), Paseo de la Sierra de Atapuerca, 3, 09002 Burgos, Spain

## Abstract

Neandertals were top predators who basically relied on middle- to large-sized ungulates for dietary purposes, but there is growing evidence that supports their consumption of plants, leporids, tortoises, marine resources, carnivores and birds. The Iberian Peninsula has provided the most abundant record of bird exploitation for meat in Europe, starting in the Middle Pleistocene. However, the bird and carnivore exploitation record was hitherto limited to the Mediterranean area of the Iberian Peninsula. Here we present the first evidence of bird and carnivore exploitation by Neandertals in the Cantabrian region. We have found cut-marks in two golden eagles, one raven, one wolf and one lynx remain from the Mousterian levels of Axlor. The obtaining of meat was likely the primary purpose of the cut-marks on the golden eagle and lynx remains. Corvids, raptors, felids and canids in Axlor could have likely acted as commensals of the Neandertals, scavenging upon the carcasses left behind by these hunter-gatherers. This could have brought them closer to Neandertal groups who could have preyed upon them. These new results provide additional information on their dietary scope and indicate a more complex interaction between Neandertals and their environment.

## Introduction

Neandertal behavioral complexity and whether their cognitive capacities parallel those present in modern humans is currently a topic of debate among archaeologists and paleontologists, and it relies on direct and indirect evidence. Neandertal behavioral complexity and flexibility is reflected in many aspects of the paleoanthropological record^1^. There is anatomical evidence consistent with spoken language, which is the basis for complex cultural transmission and abstract thinking^2–5^. This complexity can also be observed in the way Neandertals managed landscapes and settlements, including habitat structures^6–10^. In recent years we have also observed the increasing number of evidence for the practice of non-utilitarian activities, such as the burial of dead^11,12^, artistic behavior^13,14^, the elaboration and display of ornaments^15–20^, the use of pigments^21,22^ or the building up of structures such as the ring from Bruniquel^23^. From a purely technological point of view, Neandertals mastered the use of fire^24^, developed complex technological procedures, such as creating birch glue^25^ and started using bone tools more systematically^26,27^. In regard to lithic technology, several features interpreted as markers of modern behavior, such as the use of hafted tools^28^, the long distance transport of raw materials^29^, the production and use of small tools^30^, or the development of blade technologies^31^ are more common in Neandertal technology than previously thought^32^. It is worth noting that all these features may or not appear together as a bundle. In fact, it is likely that the underlying variability in these features is the result of the cultural complexity of Neandertals.

Regarding Neandertal subsistence, direct and indirect evidence of medium- to large-sized ungulate hunting in the Neandertal lineage, sometimes implying complex strategies, was present starting in the Middle Pleistocene^33–35^. Ungulates constituted the largest percentage of dietary intake of Neandertals^36–38^, which are considered as top-level predators^39,40^, though vegetables also constituted a significant component of their diet^41–43^. Additionally, there is increasing evidence showing the Neandertal exploitation of other animals for dietary purposes: leporids^44–46^, birds^47,48^, tortoises^49,50^, river fish^51^ or marine resources^52,53^.

In any case, Neandertals exploited animals, not only for dietary purposes but also for pelts, tools (e.g., retouchers), and there is increasing evidence of non-utilitarian use of animal resources in the European Middle Paleolithic record, such as mollusk and bird (talon and feather) exploitation as ornaments^16,18–20^. Thus, bird exploitation by Neandertals is an area of growing interest among researchers because it is being linked to behavioral complexity in these hunter-gatherers on a two-fold perspective: first, because bird consumption is linked to a broader diet and the capability to hunt small, fast-moving game; and second, because there is evidence of bird exploitation related to symbolic behavior.

Additionally, carnivore-human interaction during the Middle Paleolithic is also an area of interest due to its ecological implications regarding the position of Neandertals within the ecosystems they inhabited. Neandertals would have potentially competed with terrestrial carnivores for ungulate prey species^40^ and shelter^54^. There are also some examples of carnivore exploitation during the Middle Paleolithic^38,55^, but cut-marks on carnivore remains are abundant only in a few sites (Biache-Saint-Vaast, Taubach^56,57^). There are also examples of carnivore modification on Neandertal remains, which have generally been interpreted as scavenging^58–60^. Thus, any new information on human-carnivore interaction during the Middle Paleolithic provides new clues to understanding Neandertal paleoecology.

The Iberian Peninsula is a key European area for understanding the evolution^61^, paleobiology and cultural variability of Neandertals^16,53,62^. However, the few Neandertal and pre-neandertal sites/levels in the Iberian Peninsula that have yielded evidence of bird and carnivore exploitation are limited to the Mediterranean climatic zone. The recent reassessment of the faunal remains from the Axlor site (Biscay, Northern Iberian Peninsula), recovered during the excavations performed by J. M. Barandiaran between 1967 and 1974, led to the identification of three avian remains (from levels IV and V) and two carnivore remains (from levels III and V) showing anthropogenic modification. These remains were found in archeopalaeontological levels of clear Mousterian affinity. This paper reports the first evidence of bird and carnivore exploitation in the Cantabrian region and discusses its significance within the Western European context.

## Context

**Figure 1:**
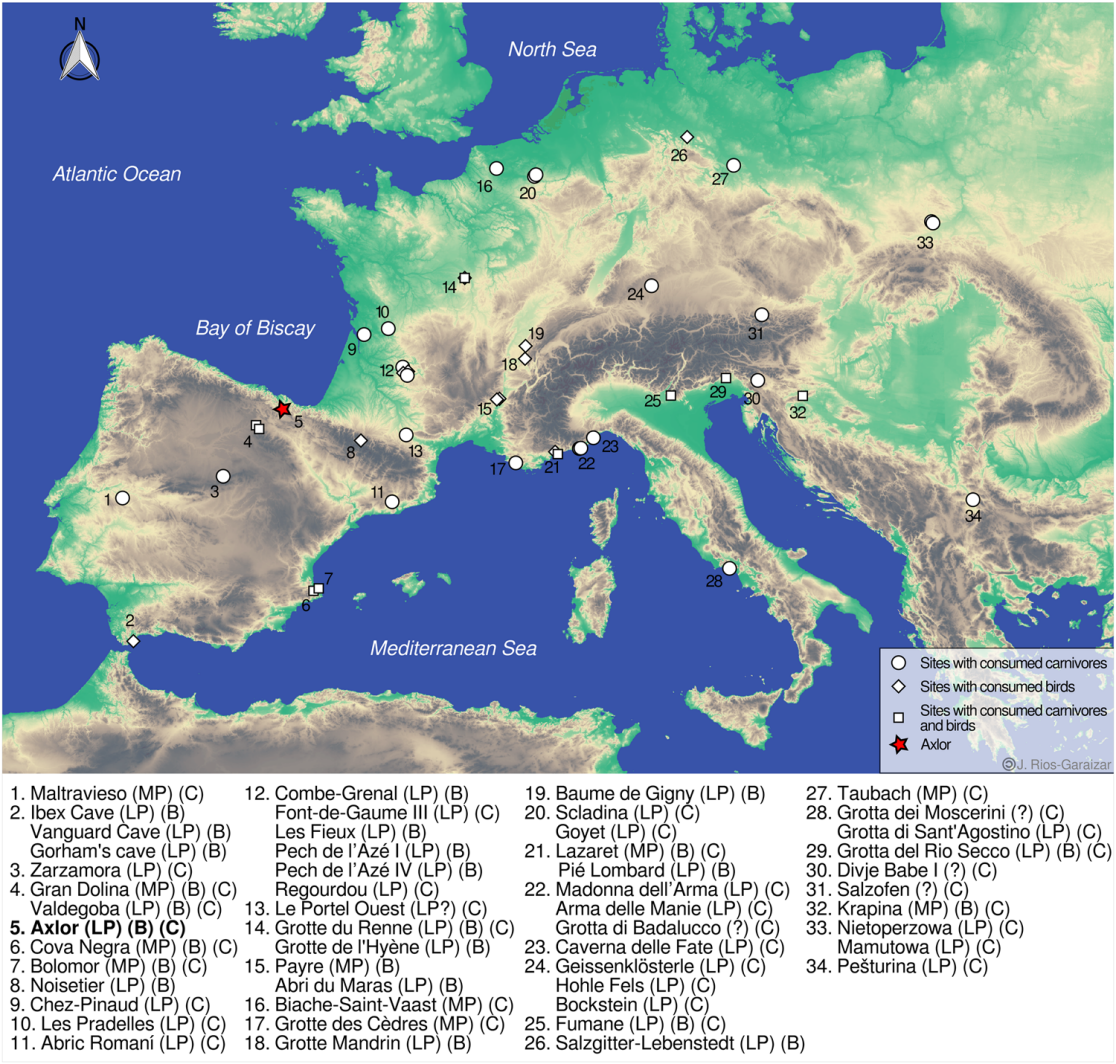
General geographic setting of Axlor with other selected Neandertal sites with bird (B) and/or carnivore (C) exploitation in Western and Central Europe. LP = Late Pleistocene; MP = Middle Pleistocene. Base cartography obtained from the European Environment Agency (Permalink; 070F2DAD-1AED-4B9B-950F-). Map generated with QGIS 2.8 Wien and Inkscape 0.91.

**Figure 2:**
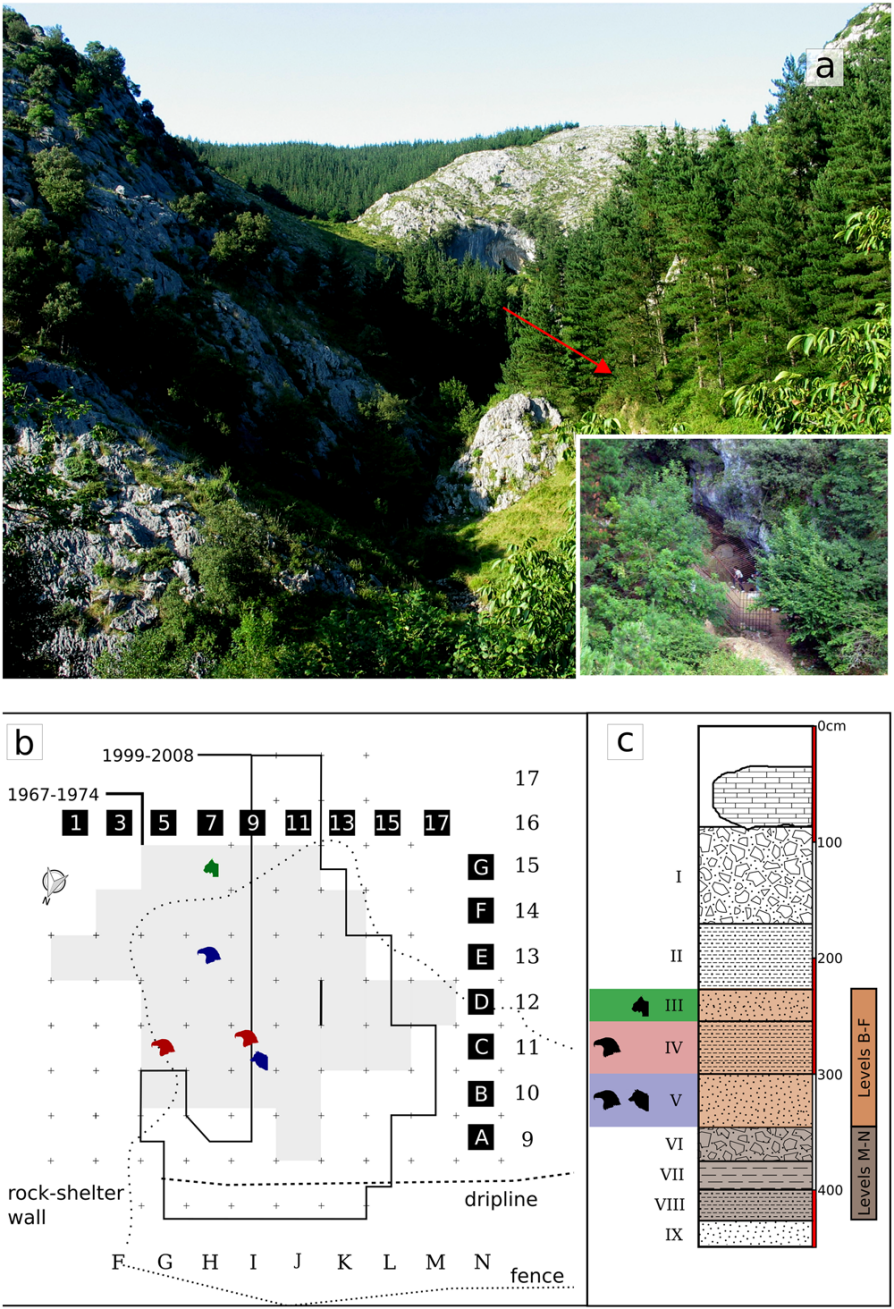
General view of the site, stratigraphy and location of the bird and remains with cut-marks within Axlor: (**a**) Lapikoerreka Valley viewed from the SW, the red arrow indicates the position of Axlor rock-shleter, in the small box is an aerial view of the rock-shelter (Photos: Joseba Rios-Garaizar); (**b**) Excavation plan, the grid system used by J. M. Barandiaran is represented by black squares and white numbers and letters, and the excavation area is shadowed in gray. The excavation area of recent excavations is marked with a thick black line, and the grid system is represented by black numbers and letters. The dotted line represents the rock-shelter wall when the site was first excavated, during the excavation this wall went back, revealing a possible cave infilling. The colors correspond to the levels to which these remains have been attributed. (**c**) Synthetic section of the 1967–1974 excavation stratigraphy, drawn from the description of the layers by J. M. Barandiaran^63^. Different levels are marked with different colors, and the presence of carnivores and birds with anthropogenic marks is represented by silhouettes. All these levels (III-V) roughly correspond to the Axlor Upper Sequence (Levels B-F of modern excavations), characterized as Charentian Mousterian Type Quina^9^.

The site of Axlor (Dima, Biscay, Basque Country) is located on the northwest slope of the Urrestei mountain, close to the Indusi stream, a tributary of the Arratia river (UTM 30 N, X: 522055.36, Y: 4774266.12, Z: 291.32; Fig. 1). Axlor was discovered in 1932 by J. M. Barandiaran while he was excavating the nearby site of Balzola. The first archaeological excavations did not take place until 1967, and encompassed a total of eight field seasons until 1974^63^, being this the last excavation being performed by this researcher. The excavations by Barandiaran unearthed a sequence composed of nine layers (I-IX), in which levels III to VIII contained Middle Paleolithic assemblages (Fig. 2). More recently, a new excavation project took place between 2000 and 2008, directed by J. E. González-Urquijo and J. J. Ibáñez Estévez (2000–2008) and also co-directed by J. Rios-Garaizar (2003–2008)^64,65^. The entire Axlor sequence was assessed during these new excavations, which added some new levels under the sequence previously excavated by Barandiaran^64^. A first essay of correlation between the Mousterian levels of the two excavations can be found in Rios-Garaizar^66^: the recent M and N basal levels are roughly correlated to Barandiaran’s basal levels (VI to VIII), while the upper levels B-F are correlated to Barandiaran’s levels III to V. There are clear differences in terms of the percentage of ungulate consumption, technological characteristics, and type of occupation of the cave between the upper and lower parts of the Mousterian sequence^66^.

The bird and carnivore remains with cut-marks have been found in levels III, IV and V. These levels have been classified as Quina Mousterian, and all of them are from the upper part of the rockshelter Mousterian sequence^66^. Five Neandertal dental remains with a maxilla fragment from the same individual (a young adult) were recovered from level III-IV^63,67^, though only three of them are curated at the Arkeologi Museoa^68^. Level IV (D in recent excavations) was initially dated to 42,010 ± 1,280 uncal BP (AMS on bone, Beta-144262)^69^ and >43,000 uncal BP (AMS on bone, Beta-225486)^70^. New ultra-filtered dates obtained from red deer with anthropogenic marks from level IV have yielded results that go beyond the radiocarbon limit (>49,300- OxA-32428; >49,900- OxA-32429)^71^, suggesting that this level is significantly older than previously thought^66^. Level V roughly corresponds to level F from recent excavations. This level yielded a date of >47,500 (Beta-225487) and another much younger one of 33,310 ± 360 (Beta-225485) that must be considered invalid^66,70^.

Levels from the upper part of the Axlor Mousterian sequence (levels III-VI from Barandiaran’s stratigraphy/levels B-F from the modern stratigraphy) represent a thick palimpsest of repeated occupations with some sterile gaps and remnants of more discrete occupations^66^. The technological features of the lithic remains in this upper part are consistent with a Quina Mousterian technocomplex. The most interesting features are the massive use of imported (>30 km far away) flint, the use of ramified strategies to assure the availability of lithic tools, and the intensive curation, production and use of lithic tools (Table S1)^66^. Interestingly, bone tools are quite abundant in this upper part of the Mousterian sequence. Among the bone tools, retouchers represent the predominant type, but other tools, such as chisels or polishers, have been also identified (Table S2)^26,72^. The faunal assemblage in these levels is dominated by red deer (*Cervus elaphus*), large bovids (*Bos/Bison*), Iberian wild goats (*Capra pyrenaica*) and, to a lesser extent, horses (*Equus ferus*) (Tables S3 and S4)^36,73^. The fauna has been described as intensively processed and as having almost no carnivore activity^73^. In fact, carnivores represent a very small percentage of the fauna^36,73^. A recent model of game procurement strategies proposes that Neandertals were not focused on local resources, suggesting that inhabitants of Axlor developed planned catchment strategies to hunt specifically-selected herbivores, which would have been scarce in the surroundings of the rockshelter^9^.

The Barandiaran faunal collection housed at the Arkeologi Museoa has two limitations. First, the excavation methodology employed by J. M. Barandiaran was not very precise. The excavation was performed using artificial spits, which produced a significant admixture of levels as the levels were not completely horizontal. While all the avian and carnivore remains belong to the upper part of the Mousterian sequence, their precise stratigraphic provenance should be approached with caution. Second, there is a bias in the representation of the faunal (and lithic) remains. In the case of the lithic assemblage, the smallest fragments or pieces made on quartz or mudstone are rarely present, while in modern excavations (2000–2008) they were significantly more abundant. The same holds true for the faunal assemblage, in which many shaft fragments and small remains were discarded. The Barandiaran collection shows a very high percentage of bones that can be taxonomically classified to a species level (c. 80%) with a clear underrepresentation of small indeterminate diaphyseal fragments. Barandiaran^63^ mentions the presence of 13,909 bone remains in level IV and 11,111 bone remains in level V. The current collection housed at the Arkeologi Museoa stores 3,762 remains from level IV and 1,616 from level V. Whether the recovery of only four avian remains in the Mousterian levels from the Barandiaran excavation is related to this bias is currently unknown. There are clear differences in terms of the percentage of ungulate consumption, technological characteristics and type of occupation of the cave between the upper and lower parts of the Mousterian sequence^36,66^. The absence of bird remains in the lowermost part of the Mousterian sequence in Axlor (levels VI-VIII) could be related to the fact that Barandiaran excavated these levels in a more restricted area.

## Data Presentation and Results

Here, we examined all the avian (NR = 18) and carnivore non-dental (NR = 92) remains from the Barandiaran collection, housed at the Arkeologi Museoa (Bilbao, Biscay), with special emphasis on those remains from the Mousterian layers.

The taxonomic classification of the avian remains from Axlor is shown in Table 1. Four remains came from the Mousterian levels IV and V, while the remaining 14 were found in level I, which has neither a clear chronology nor a cultural ascription. There are compelling taxonomic and taphonomic differences between the bird remains found at the Mousterian levels and those from level I. The bird remains from level I correspond to birds of small to medium size (e.g., starlings -*Sturnus* sp.- or common kestrel -*Falco tinnunculus-*). Half of the sample from level I show bone fractures compatible with fresh bone, with smooth surfaces and fracture orientations oblique to the main axis of the shaft. Additionally, in some cases (*n* = 9), we have detected signs of corrosion of the bone surface compatible with digestion by gastric acids (Fig. S1).

**Table 1:** Number of identified specimens (NISP) of avian remains of the Barandiaran excavations of Axlor.

Taxon	Level			Total
	I^*^	IV (Mousterian)	V (Mousterian)	
Aves indet.	2			2
Passeriformes indet.	2			2
*Sturnus* sp. (starling)	4			4
*Pyrrhocorax pyrrhocorax* (red-billed chough)	3			3
*Corvus corax* (common raven)		1**		1
*Corvus* sp.	1			1
*Falco tinnunculus* (common kestrel)	2			2
*Aquila chrysaetos* (golden eagle)		1 + 1**	1**	3
Total	14	3	1	18

^*^Indeterminate cultural ascription.

**These remains show anthropogenic marks.

**Figure 3:**
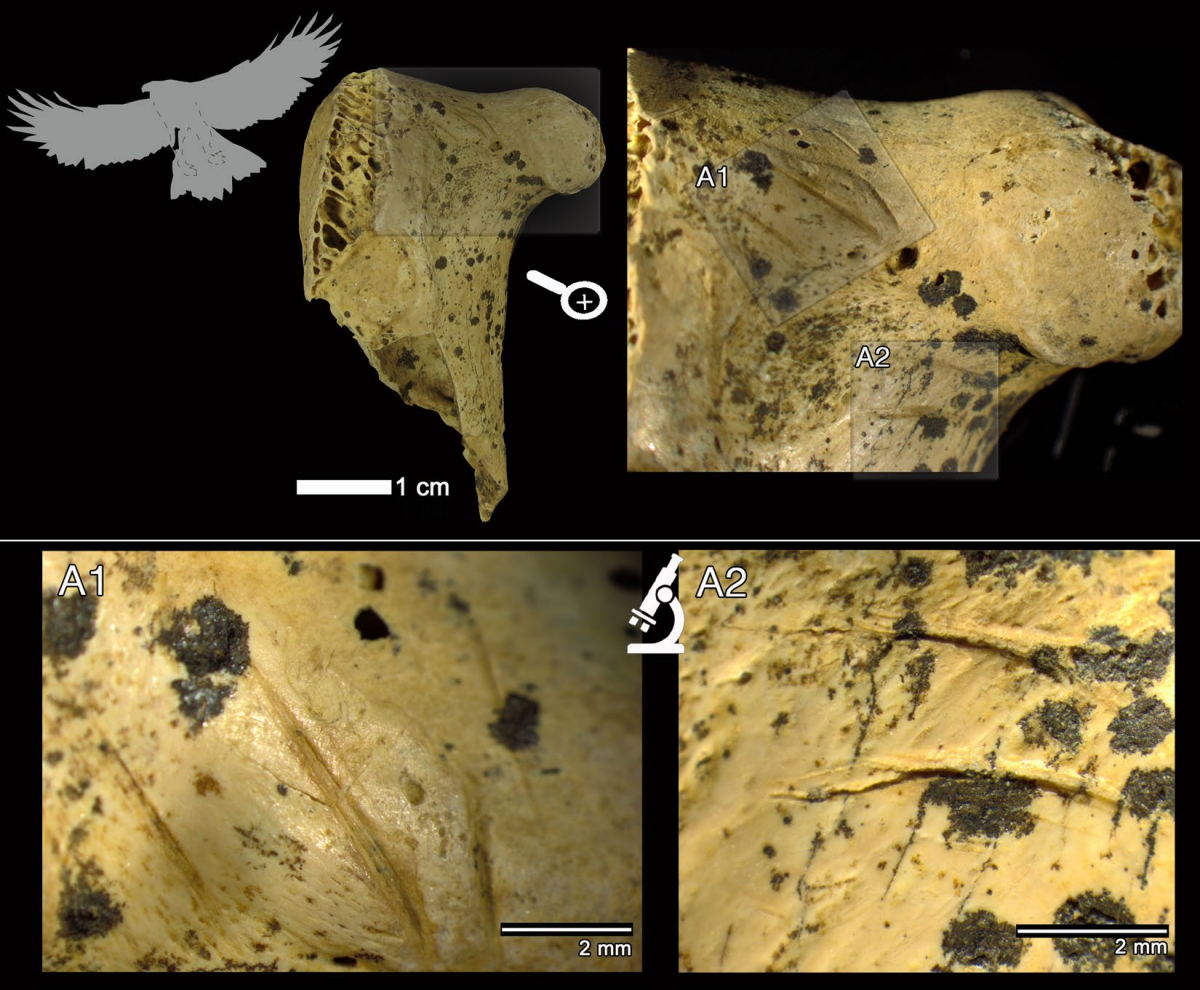
Proximal fragment of a golden eagle femur (*Aquila chrysaetos;* AX.5 C.286.153) from Axlor level IV. It is possible to observe the fracture properties of the diaphysis in the large figure. A1 and A2 indicate the two main zones with cut-marks (incisions) in the neck of the femur: (A1) lateral to the head of the femur; (A2) caudal to the head of the femur.

**Figure 4:**
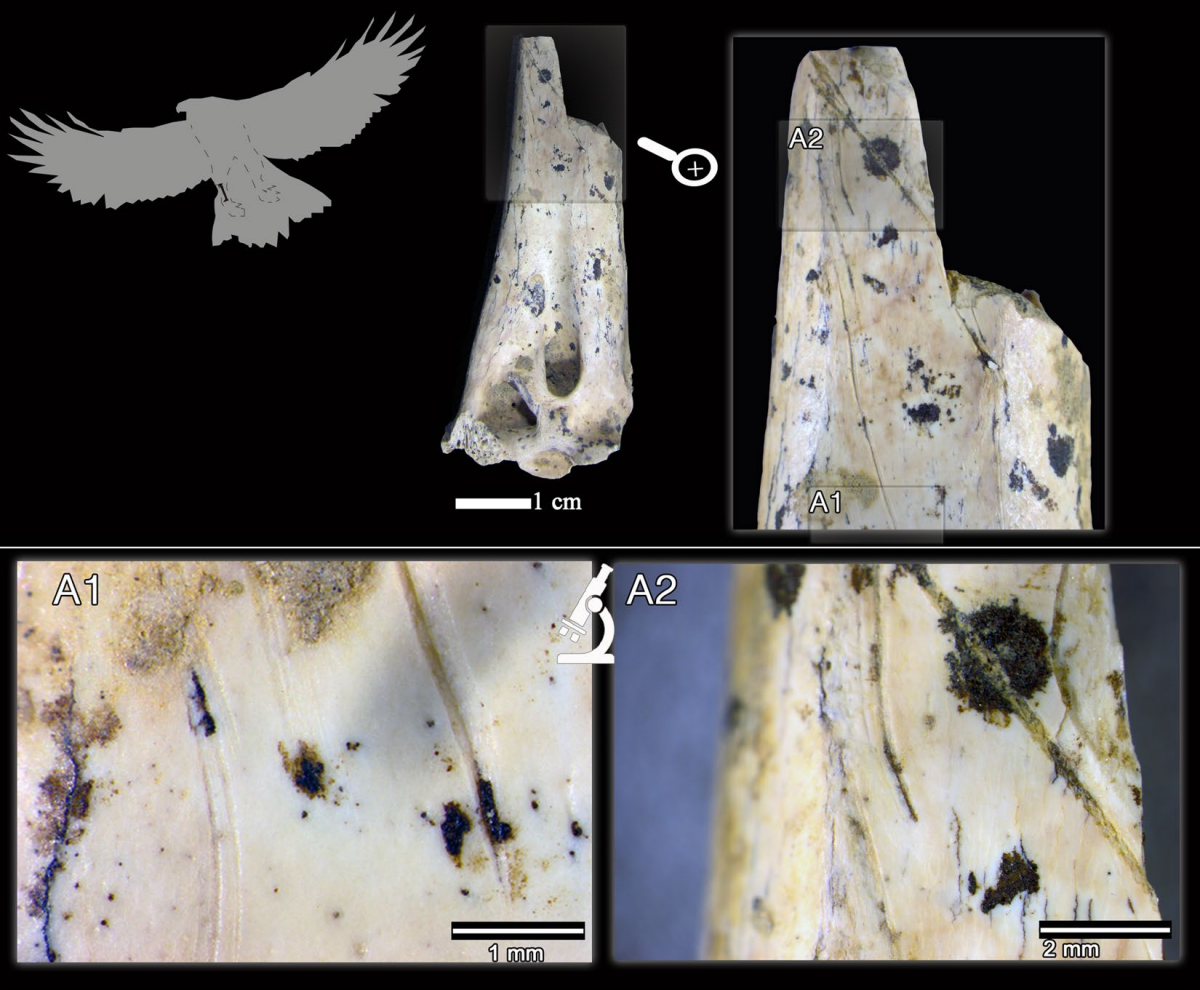
Distal fragment of a golden eagle tibiotarsus (*Aquila chrysaetos;* AX.7E.303.294, level V) where it is possible to observe both the morphology of the fracture of the diaphysis, as well as different detailed views of the cut-marks: incisions and scraping (A1, A2). All these marks demonstrate anthropic activity on this remain.

**Figure 5:**
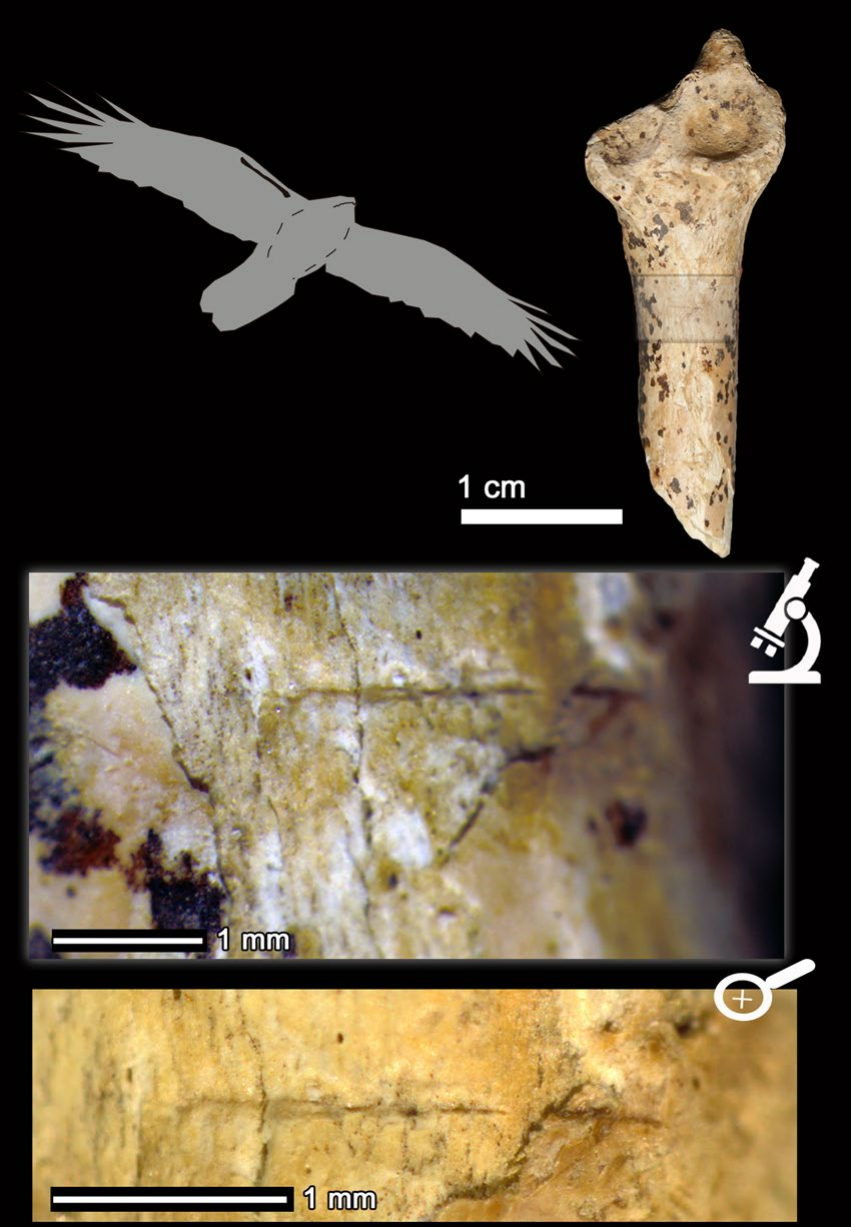
Proximal fragment of a common raven ulna (*Corvus corax;* AX.9 C.276.126, level IV), where it is possible to see a fine incision perpendicular to the long shaft of the diaphysis.

In Mousterian levels IV and V, three golden eagle (*Aquila chrysaetos*) remains and one raven (*Corvus corax*) ulnar fragment have been recovered (Fig. 2). These avian taxa are larger taxa than those represented in level I, and in three out of four cases the bones show cut-marks (Table 1). Here we describe these four remains anatomically, taxonomically and taphonomically. First, the proximal right femur of a golden eagle AX.5 C.286.153 (level IV; Fig. 3) is broken at the shaft and the oblique borders and the smooth surfaces of the breakage suggest that it was produced when the bone was still fresh. The femur neck shows six deep incisions of clear anthropogenic origin (Fig. 3). These marks were not produced by trampling and they are not tooth scores either, based on the following micro- and macroscopic observations/features: (i) the location of the marks (the neck of the femur is an area less exposed to geological agents) and, therefore, less susceptible to trampling; (ii) closed V-shape of the incisions; (iii) straight or curved trajectories, depending on the curvature of the bone surface; (iv) presence of straight and continuous microstriations located on the bottom and walls of the grooves; and (v) presence in some cases of Hertzian cones and shoulder effect (Fig. 3). The location of these marks could be related to the defleshing and dismembering of the femur. Second, the distal fragment of the tibiotarsus AX.7E.303.294 (level V; Fig. 4) of a golden eagle also shows a fracture at the shaft, which is compatible with a fresh bone fracture. Additionally, on the anterior surface of the shaft there are clear cut-marks: incisions oblique to the bone shaft and scraping marks parallel to the longitudinal axis of the bone (Fig. 4), likely related to defleshing. We cannot completely rule out that the golden eagle remains with cut-marks could belong to the same individual. The differences in depth (286 vs 303) could be related to the natural dip of the stratigraphy and their classification in different levels could be due to the excavation methodology, which did not completely respect natural layers. Third, the proximal fragment of a raven ulna (AX.9 C.276.126; level IV) shows an incision perpendicular to the longitudinal axis of the bone (Fig. 5). The surface of this bone is slightly damaged, which makes it difficult to visualize microscopic characteristics. Nevertheless, the closed V-shape of the groove, in addition to the straight trajectory, suggests an anthropogenic origin rather than trampling or carnivore modification. No fractures related to elbow over-extension^74^ have been observed in this ulna. Finally, level IV also yielded a complete phalanx of a golden eagle with no evidence of any biological surface alteration.

**Figure 6:**
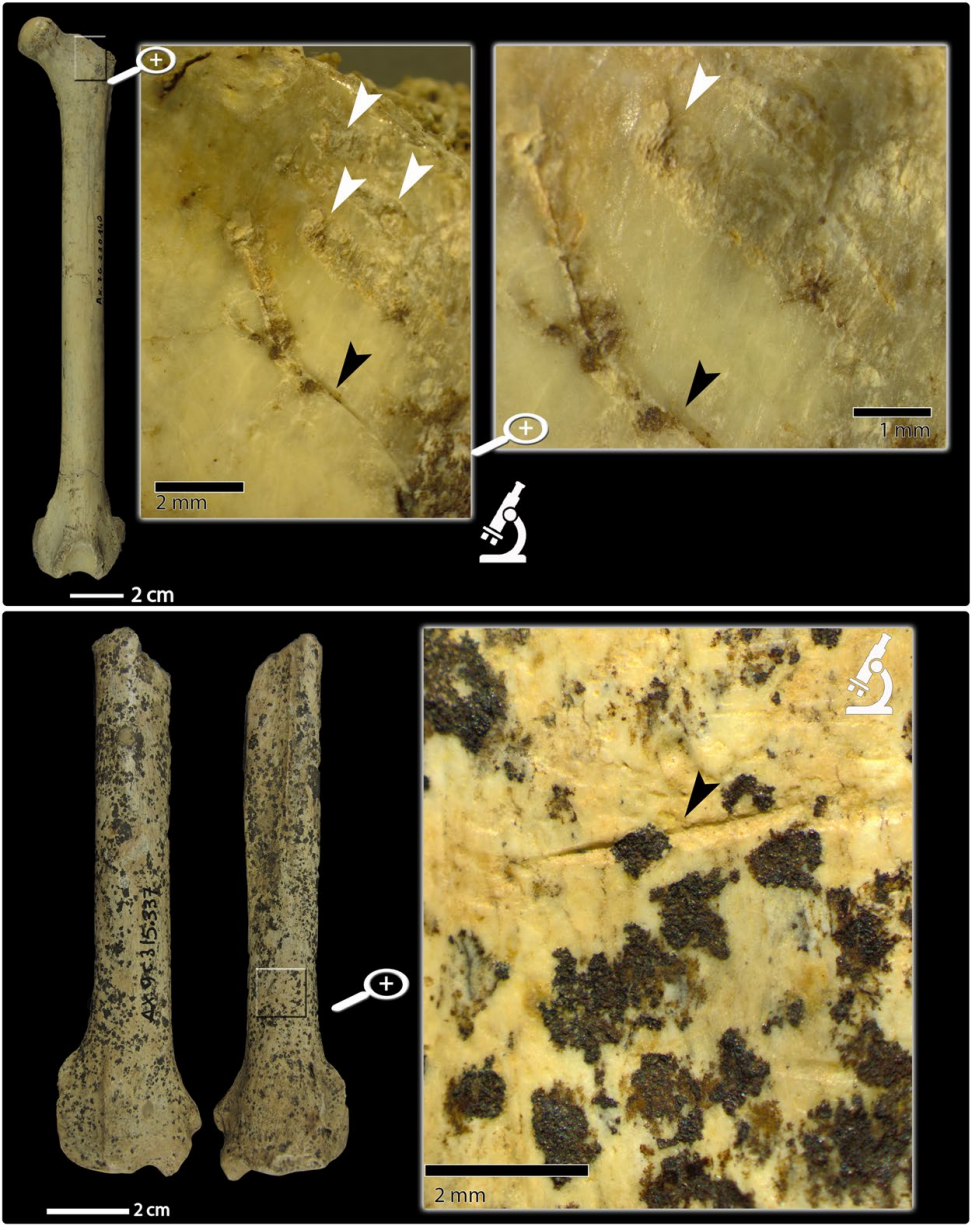
Carnivore remains with evidence of anthropogenic activity. Top: complete left lynx (cf. *Lynx* sp.) femur (AX.7 G.230.140) from level III, where it is possible to see slicing marks (black arrows) and tooth marks (pits and scores, white arrows). In the lower image it is possible to observe a slicing mark on a wolf (cf. *Canis lupus*) radius (AX.9 C.315.337) from level V (black arrow).

More than half of the analyzed carnivore remains were found in level I (Table 2), and are generally well preserved: only half of the carnivore remains present fractures of any kind, and most of them are transverse with jagged surfaces, which are related to dry bone (postmortem) fractures. Five specimens, which were discovered in levels I, V and VI (Table 2), show characteristic features associated with green bone fractures. Only two out of the 92 remains that were analyzed show evidence of cut-marks: one of them is classified as a felid and the other is classified as a canid, and both of them come from the Mousterian levels. First, AX.7 G.230.140 is a left complete femur that belongs to an adult (both epiphyses fused) felid individual left complete femur from level III (Fig. 6). This remain has been classified as cf. *Lynx* sp. This remain shows slicing marks (SL) throughout the diaphysis (Fig. 6). A series of at least 10 parallel SLs located at the lateral supracondylar tuberosity, insertion point of the gastrocnemius muscle, are likely the consequence of defleshing activities, as are the fine SLs perpendicular to the long axis of the bone present on the central part of the diaphysis. In the proximal portion, this femur displays an apparently isolated SL, diagonal to the long axis of the diaphysis (Fig. 6). Close to this SL, tooth marks (scores and pits) have been observed. In fact, one of the scores overlaps this SL (Fig. 6). The tooth marks are not abundant enough to treat them statistically, which precludes their taxonomic classification. If these marks were produced due to carnivore activity, then carnivores accessed this remain after anthropic manipulation. Second, AX.9 C.315.337 is a distal fragment of an adult (distal epiphysis fused) canid radius from level V (Fig. 6). From both a metric and morphological point of view, this specimen is more similar to wolves and, thus, it has been classified as cf. *Canis lupus* (Tables S5 and S6). On the distal part of the diaphysis, there is an isolated slicing mark (SL) (Fig. 6) with a transverse orientation in relation to the long axis of the bone. Its closed V-shape, straight trajectory, and presence of Hertzian cones allow us to rule out trampling as the origin for this incision. We interpret this slicing mark could be the result of defleshing or skinning activities.

**Table 2:** Studied carnivore non-dental NISP^a^ from Axlor (Barandiaran collection) and percentage of presence of surface alterations by level.

Surface alteration		Level (total studied NISP^a^)							
		I (55)	II (3)	III (10)	IV (15)	V (6)	VI (2)	VIII (1)	Total (92)
Physical alterations^b^	Manganese oxid coating (NISP and %)	37 (67.27%)	3 (100%)	9 (90%)	14 (93.33%)	6 (100%)	2 (100%)	1 (100%)	72 (78.26%)
Biological alterations^c^	Dissolution produced by roots (NISP and %)	8 (14.55%)	1 (33.3%)						9 (9.78%)
	Cut-marks (NISP and %)			1 (10%)		1 (16.67%)			2 (2.17%)
	Carnivore tooth marks (NISP and %)	1 (1.82%)		1 (10%)					2 (2.17%)
Fracturation type	Green bone fractures (NISP and %)	3 (5.45%)				1 (16.67%)	1 (50%)		5 (5.43%)
	Dry bone fractures (NISP and %)	6 (10.90%)		1 (10%)	6 (40%)	1 (16.67%)	1 (50%)		15 (16.3%)

^a^Number of identified specimens.

^b^None of the studied remains show evidence of weathering, crusting or dissolution.

^c^None of the studied remains show evidence of trampling or rodent activity.

We do not currently have hard evidence for the consumption of carnivore bone marrow by the Axlor Neandertals. First, none of the carnivore bones with green bone fractures show percussion marks, and second, one of the bones with cut-marks is complete. Tooth marks on carnivore remains are restricted to the femur with cut-marks from level III (Fig. 6) and to a puncture in a canid (wolf/dhole) ulna (AX.7 C.160.97) from level I (Fig. S2).

## Discussion

Axlor provides the first evidence of bird and carnivore exploitation in the Cantabrian region. Neandertals at Axlor exploited at least a golden eagle and a lynx for dietary purposes, while the purpose of the exploitation of a wolf and a raven is not yet clear. While there are an increasing number of sites with evidence of this kind of exploitation in Europe, they are still limited in absolute terms. We hypothesize that part of the reason for this absence is possibly a bias, likely due to the lack of detailed taphonomic analyses in important sequences of the Iberian Peninsula in general, and in the Cantabrian region in particular. These new findings significantly expand the observed geographical range of Late Pleistocene Neandertal bird and carnivore exploitation in Western Europe (Fig. 1).

### Axlor bird exploitation in the Iberian and Western European context

Neandertal bird exploitation seems to have a dual purpose: meat consumption and feather and talon exploitation to be used for non-utilitarian purposes (e.g., ornaments^16,18,19^; Table S7). It should be noted that the Iberian Peninsula has provided most of the evidence of meat consumption of birds by Neandertals^47,48,75^, starting in the Middle Pleistocene^75–77^ (Table S7). The Iberian Peninsula has also yielded evidence of bird exploitation to obtain feathers^16^ but yet no evidence of talon exploitation has been found, which seems to be currently limited to France, Italy and Croatia^17,19,78–82^. Regarding the consumption of birds, in the Iberian Peninsula, pigeons and choughs were the most exploited species for dietary purposes during the Late Pleistocene^47,48^ (Table S7). However, cut-marks on several raptor anatomical elements cannot directly be linked to feather and/or talon exploitation^16^ and thus in Gorham’s cave the exploitation of raptors for meat consumption was not exceptional. Axlor provides an additional example of raptor remains for meat consumption, in this case a golden eagle, which has a direct parallel in the golden eagle femur from Les Fieux, France^83^. In summary, the evidence from Axlor reinforces the potential of raptors for meat exploitation and not only for talon and feather exploitation^19,82^.

On the other hand, birds with dark remiges seem to be overrepresented in Mousterian levels^16^. The exploitation of ulnae in corvids could be related to the removal of the secondaries (feathers) and the experimentation in large raptors to exploit these feathers results in cut-marks of diverse orientation^84^. The fact that only one broken raven specimen (a partial ulna), with a sole cut-mark, has been found in Axlor precludes us from inferring the purpose of this bird’s exploitation, though neither meat consumption nor feather exploitation can currently be ruled out. Raven exploitation by Neandertals is currently limited to Les Fieux for dietary purposes^80^ and Zaskalnaya VI has provided a raven radius with a series of cut-marks, which have been interpreted as symbolic behavior^85^.

The absence of additional evidence of bird exploitation in the Cantabrian region could merely be due to a lack of more taphonomic analyses on bird remains. In important Mousterian sequences, such as El Castillo, taxa exploited for meat consumption (e.g., choughs -*Pyrrhocorax* sp., also present in El Conde), as well as taxa used for talon exploitation as a potential symbolic/decorative behavior by Neandertals (e.g., cinereus vulture- *Aegypius monachus*), have been recovered. However, no detailed taphonomic analysis has been carried out and no anthropogenic modification of these remains has been detected so far^86^. In close proximity to Axlor, the site of Amalda (Zestoa, Gipuzkoa) has yielded a total of 28 bird remains in the Mousterian level (level VII)^87^. From the eight taxa represented, there is a presence of corvids (*Corvus* and *Pyrrhocorax*) and raptors (*Aquila chrysaetos*), to name a few, which have been exploited in Axlor and elsewhere. Finally, in other sites of the Cantabrian region, where important Mousterian sequences have been excavated (e.g., Arrillor, Covalejos, Esquilleu, Morín, El Cuco), there is still no information regarding whether avian remains have been recovered, and if so, whether their accumulation is anthropogenic. Therefore, it is likely that future studies will provide new evidence of bird exploitation in the Cantabrian region. However, it is also likely that, due to ecological restraints, the evidence will be more scarce than in sites like Gorham’s cave, located in a biodiversity hotspot, especially with regard to bird ecology^88^.

### Axlor carnivore exploitation in the Iberian and Western European context

Carnivore remains associated with Middle Paleolithic contexts with cut-marks or other anthropogenic alterations are scarce in the Iberian Peninsula (Table S8) and are normally interpreted as opportunistic and isolated episodes^55^. The earliest evidence of carnivore exploitation in the Iberian Peninsula comes from the Middle Pleistocene: Gran Dolina TD10.1 (Burgos; MIS 9) and has yielded several lion (*Panthera leo fossilis*) and one fox (*Vulpes vulpes*) remains with evidence of anthropogenic manipulation (cut marks, fresh fractures) for the obtaining of food^77,89^. In the Sala de los Huesos at Maltravieso (Cáceres), three skeletal elements from a spotted hyena (*Crocuta crocuta*) dating to the end of the Middle Pleistocene show evidence of butchering^90,91^. Cova Negra (Valencia) has yielded a dhole mandible (*Cuon* cf. *alpinus*) with marks made by lithic industry^92^ and a thermoaltered 5^th^ metatarsal from a leopard (*Panthera pardus*), which shows cut-marks related to skinning^93^. The Middle Paleolithic site of Valdegoba has yielded two canid and an additional carnivore remain with anthropogenic marks^94^. The Middle Paleolithic levels of Zafarraya have also provided evidence of anthropic action on carnivore dental remains: a fractured *Ursus* canine and burnt *Ursus* and *Lynx* canines^95^. Level IV of Bolomor has yielded evidence of anthropogenic manipulation of several carnivores including lion, fox and lynx for both meat and pelt obtention^75^. Sima de las Palomas has also yielded a burnt leopard bone and two articulated leopard paws close to SP-92 and SP-97 and slightly below SP-96 Neandertal individuals^96^. Level O of Abric Romaní (Barcelona) yielded a partial wildcat skeleton with cut-marks interpreted as the result of skinning and the obtaining of food^55^. Finally, the Cueva de la Zarzamora site has yielded a lynx (*Lynx* sp.) humerus with cut-marks from a hyena den context, which has a chronology (>44 ka BP)^97^ that would be consistent with a Neandertal presence in the zone.

Neandertal carnivore exploitation seems to have mainly two aims: meat consumption and hide obtention, and seems to be the case in the limited record from the Iberian Peninsula (Table S8). However, carnivore bones were also used as retouchers during the Middle Paleolithic in Scladina^98^, Caverna delle Fate^99^ and Fumane^100^ and there is an intriguing example of a possible example of numeric notation on a spotted hyena femur from Les Pradelles^101^. In the latter case, whether the fact that the object in which these “notations” were done being a carnivore remain was purposefully sought is currently unknown. However, the presence of the articulated leopards paws close to Neandertal individuals in Sima de las Palomas^96^ could mean that in some Neandertal groups, certain carnivores may have had a symbolic role. In any case, in the case of Axlor, the available evidence at hand is consistent with the interpretation that in Europe, carnivore exploitation by Neandertals mainly focused in meat and pelt obtention (Table S8).

In any case, carnivore exploitation during the Middle Paleolithic of Europe is, similar to the case of bird exploitation, a rare event in terms of number of sites with this kind of evidence (Table S8) and the Iberian Peninsula is no exception to this pattern. This exploitation may represent an occasional access in order to obtain mostly meat and hides (Table S8). However, two sites, Biache-Saint-Vaast and Taubach, have provided a large amount of bones with cut-marks, that could represent a systematic exploitation of different species of bears^56,57^. In fact, different species of bears were the most exploited species for both meat and pelts, but their bones were also used as tools -*retouchoirs*- (Table S8)^100^. Surprisingly, no bear with cut-marks has been found in the Middle Paleolithic Iberian record, which could be a sampling bias. It is possible that the current evidence for Middle Paleolithic dhole exploitation is limited to the Iberian Peninsula^75^ for the same reason.

### Bird and carnivore exploitation in the context of the Cantabrian region

Evidence of bird and carnivore exploitation in Axlor has been found in the upper part of the Mousterian sequence. The faunal assemblage recovered at these upper layers indicates that red deer, large bovids, wild goats and, to a lesser extent, horses, were the main component of the diet of these Neandertals^36,73^ (Tables S3 and S4). Nevertheless, here we demonstrate that Neandertals in the Cantabrian region could have also exploited birds and carnivores for dietary purposes. Within the upper part of the Mousterian sequence, some levels (D and F from the modern excavations) are thick palimpsests, formed due to repeated occupations of the site, while the lower density of findings in other levels (e.g. B-C)^73^ suggest more ephemeral occupations^66^. The excavation of level D has yielded a large number of bone retouchers (Table S2), which indicates that certain bones were preferentially selected, both anatomically and taxonomically, from those available as a by-product of ungulate hunting^72^. Within this Upper Mousterian sequence, the excavation of level IV by Barandiaran yielded the largest number of lithic remains (Table S1), which is consistent with the anthropogenic nature of these occupations. It is likely that the scarce number of bird and carnivore remains with anthropogenic activity is likely independent of the intensity of occupation and the Axlor case constitutes likely opportunistic behavior based on punctual prey availability. In fact, compared to the rich ungulate assemblage found in Axlor, the ecological impact on the carnivore and bird populations would have been negligible. Assuming that it was an occasional behavior, more intense occupations with a larger number of paleontological findings can provide more evidence of carnivore and bird exploitation. This cannot currently be tested in the Barandiaran collection due to the bias in the recovery/preservation of the paleontological material and should be tested in the paleontological and taphonomic analysis of the faunal remains from the modern excavations.

The evidence from Axlor adds to the still scarce evidence of exploitation of marine and plant resources in the Cantabrian region. The Mousterian layers of El Cuco rock-shelter have yielded a significant amount of limpets from genus *Patella*, and marginal amounts of other mollusks (*Ocenebra erinaceus*, *Acanthocardia* sp., *Gibbula* sp.), along with the sea urchin *Paracentrotus lividus*. In any case, macromammal remains would still constitute the majority of the caloric intake of the Neandertals in this site^52^. The dental calculus from Sidrón Neandertals indicates the consumption of mushrooms, pine nuts, and moss, and the fact that some of the carbohydrates (perhaps the nuts) were being cooked, and surprisingly, no trace of animal consumption was found^102,103^.

Corvids, raptors, felids and canids in Axlor could have likely acted as commensals of Neandertals, scavenging upon the carcasses left behind by these hunter-gatherers. This could have brought them closer to Neandertal groups, who could have preyed upon them. In fact, a recent study demonstrates a clear relationship between raptors and corvids, which are regular scavengers, and Neandertals^104^. Furthermore, preying upon carnivores would have provided Neandertals with meat and pelts and, as a side-effect, would result in the elimination of ecological competitors in the case of medium to large-sized predators.

Axlor provides the first evidence of carnivore and bird consumption for the Cantabrian region and is one of the very few examples found in the Iberian Peninsula for this kind of exploitation. While limited and likely opportunistic, this evidence, together with that of nearby sites, implies a much broader diet for Neandertals inhabiting the Cantabrian region than what was previously thought.

When we look to the European Middle Paleolithic record of carnivore and bird exploitation (Tables S7 and S8), there seems to be a pattern in the taxonomic choice or preference of the prey species and the objective of this exploitation. The Axlor bird exploitation evidence is consistent with meat exploitation, which mainly occurs on the Iberian Peninsula. The carnivore exploitation in Axlor is currently limited to a felid and a canid, which are the most common taxa in the Iberian Peninsula, while at a European level bears are the most exploited taxon by Neandertals (Table S8). During the Middle Paleolithic, Cave bears (*Ursus spelaeus*) were limited to the north and center of the Iberian Peninsula^105,106^ while brown bears (*U. arctos*) were present throughout the whole Iberian Peninsula^73,77,107^. At the same time, felids and canids were present throughout Europe (Table S8)^100,108^ and thus, despite ecological particularities between the Iberian Peninsula and the rest of Europe, a priori both bears and canids were inhabiting the whole Europe. In the case of dholes, the only remains with cut-marks have been found in the Iberian Peninsula (Table S9). This is also likely due to fact that the dhole fossil sample is more scarce compared to that of wolves, and that dholes have not been studied so intensively, and thus, it is likely that in cases in which isolated dentition and postcrania has been found, they have been directly assigned to *Canis lupus*^75^. We hypothesize that these perceived differences in the taxon selection in the carnivore exploitation, i.e. more focused on felids and canids in the Iberian Peninsula and more focused in bears in the rest of Europe, could reflect the cultural variability of Neandertal populations throughout Europe during the late Middle and Upper Pleistocene. However, sampling bias cannot currently rule out and additional studies on Middle Paleolithic carnivore remains are necessary to test this hypothesis, as traditionally, taphonomic studies on faunal remains in Middle Paleolithic sites were more focused on the ungulate remains. In summary, the fossil remains from Axlor presented here add to the growing corpus of evidence of behavioral versatility of Neandertals, especially in regard to the exploitation of animal resources.

## Methods Summary

The bird remains were anatomically and taxonomically identified using both bibliography and reference collections (Museo de Ciencias Naturales de la Universidad de Zaragoza, University of Ferrara, and the Muséum national d’Histoire naturelle of Paris). For the birds taxonomic identification, different keys were used^109–115^. For the analysis of the paleontological record of the species, the works of Mlíkovský^116^ and Tyberg^117^ were used. The anatomic and taxonomic assessments of the carnivore remains were conducted using standard osteological atlases^118^ as well as modern and fossil samples housed in different institutions (UCM-ISCIII; Arkeologi museoa; Gordailua). The canids from Axlor were also compared to an extant *Cuon alpinus* sample, housed at the Museo Anatómico (Universidad de Valladolid), dhole fossils, housed at the Museu de Prehistòria de València, and extant wolf (*Canis lupus*) specimens, housed at the Estación Biológica de Doñana (Sevilla) and the Museo Nacional de Ciencias Naturales (Madrid).

An Olympus SZX10 (stereoscopic zoom microscope) was used to examine surface modification on bone remains. The following taphonomic parameters were studied: physical alterations, biological alterations and fracturation type. The studied physical alterations were the weathering, presence/absence of dissolution, crusting, and manganese oxides.

The studied biological alterations included: dissolution marks produced by roots, trampling marks, as well as anthropogenic, carnivore, and rodent activity. The studied anthropogenic modifications included marks produced by lithic industry (slicing marks, scrape marks and chop marks), and other anthropogenic activity (human tooth marks), following Rodríguez-Hidalgo^34^, Sala and Conard^119^, Landt^120^ and references therein. For the cut mark analysis, different microscopic characteristics were taken into account: number, location and orientation of the marks; shape and trajectory of the incisions; presence, trajectory and location of microstriations; presence of Hertzian cones, shoulder effects and barbs^74,121,122^. For the differentiation between cut marks and trampling we have used the methodology proposed by Domínguez-Rodrigo and colleagues^123^.

The studied carnivore activity includes that produced by carnivore teeth and gastric acids^124^. Carnivore tooth marks on bone surfaces were classified into pits, punctures, furrowing, scores and dissolution due to gastric acids^124–128^.

Finally, the study of the bone breakage pattern was focused mainly on the long bones following the previously-proposed criteria^74,129,130^ in terms of: fracture outline, fracture angle, fracture edge, shaft circumference and shaft fragment. Previous studies have demonstrated that long bones with transverse fractures to the long axis, complete circumferences and fracture edges with right angles and jagged surfaces are commonly associated with dry bone fractures (which occur postmortem). Conversely, oblique fractures with bevelled angles of the fracture plane, incomplete circumferences and smooth surfaces are commonly associated with fresh or green bone fractures (perimortem)^129,130^.

## Electronic supplementary material

Supplementary Information
