# Supplementary material for: First data of Neandertal bird and carnivore exploitation in the Cantabrian Region (Axlor; Barandiaran excavations; Dima, Biscay, Northern Iberian Peninsula)
Source: Sci Rep. 2018 Jul 12;8:10551. doi: 10.1038/s41598-018-28377-y (PMC6043621; doi:10.1038/s41598-018-28377-y)
Supplement: Supplementary file 1 — Supplementary Information [file 41598_2018_28377_MOESM1_ESM.pdf]

First data of Neandertal bird and carnivore exploitation in the Cantabrian Region (Axlor; Barandiaran excavations; Dima, Biscay, Northern Iberian Peninsula)

Asier Gómez-Olivencia[1,2,3*]
Nohemi Sala[3]
Carmen Núñez-Lahuerta[4]
Alfred Sanchis[5]
Mikel Arlegi[1,6]
Joseba Rios-Garaizar[7]

Supplementary Information

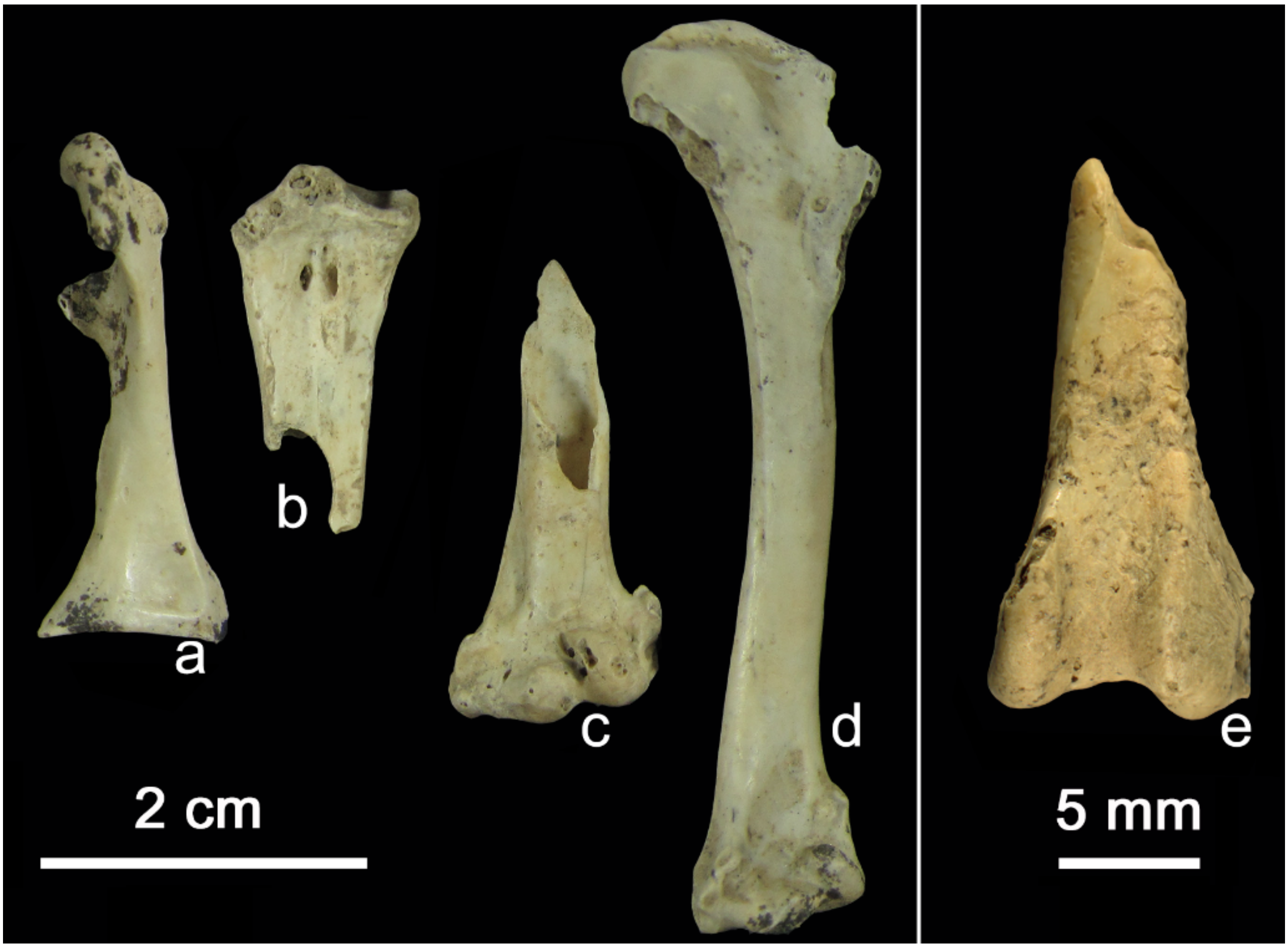

Figure S1. Bird remains from level I of Axlor: a) common kestrel (*Falco tinnunculus*): complete left coracoid bone; b) Aves indet.: proximal fragment of a tarsometatarsus; c) red-billed chough (*Pyrrhocorax pyrrhocorax*): distal half of a left humerus; d) common kestrel (*Falco tinnunculus*): complete humerus; e) corvid (*Corvus* sp.): distal fragment of a femur. Some of these bones (b, c, e) show the characteristic pattern of fresh-bone fracture. Additionally, in (e) it is possible to observe the corrosion on the surface of the bone which could be due to gastric acid action.

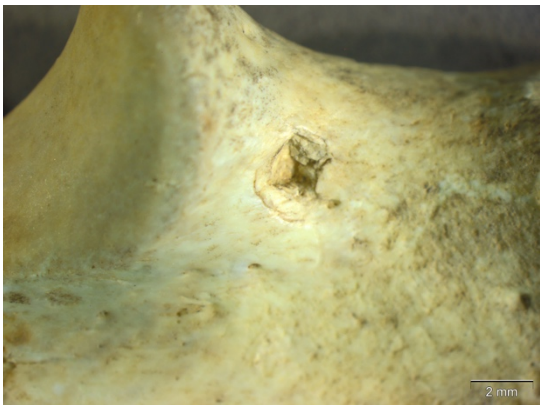

Figure S2. Detail of the puncture on a canid (*Canis/Cuon*) ulna (AX.7C.160.97) from level I.

Table S1
Number of lithic remains per level from Barandiaran's excavations in Axlor housed at the Arkeologi Museoa (Bilbao)

Level	Number of lithic remains
III	1,059
IV	13,086
V	2,975
VI	1,375
VII	208
VIII	266

Table S2
Number of bone retouchers from level D[a] of Axlor and their taxonomic assignments compared to the percentage of red deer and large bovid long bone representation.[1]

<table>
<tr><th rowspan="2">Anatomical region</th><th colspan="6">Bone retouchers from level D</th><th colspan="2">% of the long bone representation</th></tr>
<tr><th>Large bovid</th><th>Cervus elaphus</th><th>Capra pyrenaica</th><th>Small herbivore</th><th>Large herbivore</th><th>Indet.</th><th>Large bovid</th><th>Cervus elaphus</th></tr>
<tr><td>Rib</td><td>2</td><td></td><td rowspan="7">1</td><td rowspan="7">12</td><td rowspan="7">43</td><td rowspan="7">12</td><td>-</td><td>-</td></tr>
<tr><td>Humerus</td><td>1</td><td>2</td><td>25%</td><td>25%</td></tr>
<tr><td>Radius</td><td>1</td><td>2</td><td>10%</td><td>3%</td></tr>
<tr><td>Femur</td><td>4</td><td>3</td><td>15%</td><td>8%</td></tr>
<tr><td>Tibia</td><td>15</td><td>13</td><td>31%</td><td>25%</td></tr>
<tr><td>Metapodial</td><td>7</td><td>24</td><td>19%</td><td>39%</td></tr>
<tr><td>Indeterminate long bone</td><td>10</td><td>3</td><td>-</td><td>-</td></tr>
<tr><td>Total</td><td>40</td><td>47</td><td>1</td><td>12</td><td>43</td><td>12</td><td>100%</td><td>100%</td></tr>
</table>

[a] Field seasons: 1999-2003.

Table S3

Number of identified specimens (NISP) and percentage regarding the total found in levels B-C and D of the recent excavations of Axlor.[2,a]

Level	B-C		D	
**Taxon**[b]	**NISP**	**%**[b]	**NISP**	**%**[b]
Equus ferus	31	20.53	42	5.31
Bos/Bison	41	27.15	258	32.62
Capra pyrenaica	41	27.15	204	25.79
Rupicapra pyrenaica	1	0.67	7	0.88
Cervus elaphus	32	21.19	279	35.27
Capreolus capreolus	3	1.99		
Sus scrofa			1	0.065
Vulpes vulpes	2	1.32		
Panthera pardus			1	0.065
Total (ungulates)	149	98.68	790	99.935
Total	151	100.00	791	100.00

[a] The faunal list includes four *Stephanorhinus hemitoechus* remains[2]. However, this is an error (Castaños, personal communication).

[b] According to Castaños (2005: 204)[2]: "La presencia de Mamíferos no Ungulados es casi inexistente. Si a ello se añade el patrón de fragmentación de los huesos con un claro predominio de fragmentos de diáfisis de huesos largos, parece que nos encontramos ante una muestra de origen exclusivamente antrópico similar a las de Covalejos y Arrillor.". Approximate translation: There is almost no presence of non-ungulate mammals. Additionally, the fragmentation pattern of the bones shows a clear dominance of long bone shafts. These two data suggest that this sample is exclusively of anthropogenic origin, similar to the samples of Covalejos and Arrillor).

Table S4

Number of identified specimens (NISP) and percentage regarding the total NISP from the macro-faunal remains recovered during Barandiaran's excavations[3] from the Mousterian levels of Axlor.[4]

Taxon	III		IV		V+VI		VII		VIII	
	NISP	%	NISP	%	NISP	%	NISP	%	NISP	%
Equus ferus	41	30.60	72	12.18	23	3.82			3	1.84
Bos/Bison	35	26.12	171	28.93	61	10.13	3	5.36	2	1.23
Capra pyrenaica	38	28.36	193	32.66	117	19.44	22	39.29	23	14.11
Rupicapra pyrenaica	1	0.75	12	2.03	75	12.46	8	14.29	13	7.98
Cervus elaphus	18	13.43	137	23.18	316	52.49	23	41.07	120	73.62
Capreolus capreolus					1	0.17			1	0.61
Rangifer tarandus	1	0.75	1	0.17						
Sus scrofa					1	0.17			1	0.61
Ursus spelaeus			3	0.51	1	0.17				
Meles meles			1	0.17						
Canis lupus			1	0.17	3	0.50				
Vulpes vulpes					3	0.50				
Lynx sp.					1	0.17				
Total (ungulates)	134	100.00	586	99.15	594	98.67	56	100.00	163	100.00
Total	134	100.00	591	100.00	602	100.00	56	100.00	163	100.00

Table S5
Distal breadth of the canid radius from Axlor (in mm), attributed to a wolf (cf. *Canis lupus*), compared to extant and fossil dholes and extant *Canis lupus*.

**Taxon**	**Site/Label**	**Radius distal breadth**[a]	**Reference**
cf. *Canis lupus*	Axlor (right)	-/31.5	This study
Cuon alpinus (fossil)	Parpalló (left)	29.3/-	[5]
	Obarreta (left/right)	25.5/25.0	[6]
	Trinchera Galería (left/right)	24.0/24.5	[5]
Cuon alpinus (extant)	7106	24.0/24.1	Sanchis, unpublished data
	6764	24.0/23.2	Sanchis, unpublished data
	6784	22.5/22.6	Sanchis, unpublished data
	6765	23.1/22.8	Sanchis, unpublished data
	6412	22.8/22.5	Sanchis, unpublished data
Canis lupus (*n* = 9; extant)	Mean ± SD	31.7 ± 1.87	[5]
	Range	28.2-34.0	[5]

[a] Two entries refer to left/right.

Table S6
Morphological characters of the radius that distinguish *Canis lupus* from *Cuon alpinus*.

**Criterium**	**Reference**	**Axlor**	***Cuon alpinus***	***Canis lupus***
Medial border of the diaphysis	Sanchis *et al*., manuscript in preparation	Acute, presence of a crest	More rounded	More acute, presence of a crest
Lateral edge of the distal articulation	Sanchis *et al*., manuscript in preparation	Straight-like	Curved	Straighter
Styloid process	[5]	Long and pointed	Shorter, less angular and blunter	Longer and pointed
Palmar surface of the distal half of the diaphysis	Sanchis *et al*., manuscript in preparation	Flat	More curved	Flatter
Development of the lateral part of the distal epiphysis	Sanchis *et al*., manuscript in preparation	Very developed	Less developed	More developed

Table S7

List of European Mousterian sites with evidence of cut-marks on bird bones.

Site		Level/Unit	Chronology	Taxon	Common name	Element(s)	NISP with cut-marks	Interpretation	Reference
**Iberian Peninsula**									
	Axlor	IV	MIS 3	*Aquila chrysaetos*	Golden eagle	Femur	1	Defleshing	This study
		IV	MIS 3	*Corvus corax*	Common raven	Ulna	1	?	This study
		V	MIS 3	*Aquila chrysaetos*	Golden eagle	Tibiotarsus	1	Defleshing	This study
	Valdegoba		MIS 3-5	Aves indet.	Indeterminate bird		1		[7]
	Ibex cave		53.9-34.8 ka	*Pyrrhocorax pyrrhocorax*	Red-billed chough	Femur and tarsometatarsi	4	Defleshing	[8]
	Vanguard cave		50-40 ka	*Gyps fulvus*	Griffon vulture	Ulna	2	Defleshing	[8]
				Pyrrhocorax pyrrhocorax	Red-billed chough	Humerus	1	Defleshing	[8]
	Gorham's Cave							Symbolic (feathers), Defleshing	[8-10]
		IV	c. 50-27.9 ka	*Columba*[1]	Pigeon	Several elements	16		[9,10]
				Pyrrhocorax graculus	Alpine chough	Humerus and ulna	9		[8,10]
				Pyrrhocorax pyrrhocorax	Red-billed chough	Humerus and ulna	6		[8]
				Aquila chrysaetos	Golden eagle	Ulna	1		[8]
				Gyps melitensis/fulvus	Griffon vulture	Femur	1		[8]
				Milvus migrans	Black kite	Tibiotarsus	1		[8]
				Milvus milvus	Red kite	Several elements	4		[8]
		BeSm (PLSsm).3		*Columba*[a]	Pigeon	Humerus and femur	2		[9,10]
				Pyrrhocorax pyrrhocorax	Chough	Carpometacarpus	1		[10]
		LBSmcf.2		*Columba*[a]	Pigeon	Several elements	5		[9,10]
		LBSmcf.4		*Columba*[a]	Pigeon	Humerus and tarsometatarsus	2		[9,10]

				Corvus/Pica[c]	Corvid	Humerus	1		[10]
				Pyrrhocorax[b]	Chough	Carpometacarpus	1		[10]
		LBSmcf.8		*Columba*[a]		Ulna	1		[9,10]
		LBSmcf.9		*Pyrrhocorax*[b]	Chough	Ulna	1		[10]
		SSLm(Usm).3		*Pyrrhocorax*[b]	Chough	Humerus	1		[10]
		SSLm(Usm).5		*Columba*[a]	Pigeon	Femur and humerus	2		[9,10]
				Corvus/Pica	Corvid	Ulna	1		[10]
	Cova Negra	IIIb	MIS6-beginning of MIS5	*Columba livia/oenas*	Rock/stock dove	Several elements	29	Defleshing	[11]
				Pyrrhocorax/Corvus[d]	Corvids	Several elements	26	Defleshing	
				Alectoris sp.	Partridge	Femur	1	Defleshing	
				Falco naumanni	Lesser kestrel	Scapula		Defleshing	
				Coracias garrulus	Roller	Coracoid		Defleshing	
				Fringillidae	Finches	Humerus and ulna	4	Defleshing	
				Turdus merula	Common blackbird	Ulna	1	Defleshing	
				Ptyonoprogne rupestris	Crag martin	Humerus	1	Defleshing	
				-	Unidentified species	Synsacrum, humerus, phalanx	3	Defleshing	
	Bolomor	IV	>120 ka BP	Passeriformes		Fibula	1	Defleshing	[12]
				Corvidae		Several elements	4	Defleshing	
				Galliformes		Femur and tibiotarsus	2	Defleshing	
				Phasianidae		Ulna and carpometacarpus	2	Defleshing	
				Columba sp.	Pigeon	Several elements	7	Defleshing	
				Anas sp.	Dubbling duck	Several elements	5	Defleshing	
				Aythya sp.	Diving duck	Several elements	8	Defleshing	
				-	Unidentified bird	Long bones	3	Defleshing	

		XI	152 ± 23 ka	*Aythia* sp.	Diving duck	Several elements	18	Defleshing	[13]
		XII	180 ka	*Cygnus olor*	Mute swan		4		[14]
		XVIIa	MIS 9	Galliformes		Coracoid	1	Defleshing	[15]
				Phasianidae		Ulna, femur	2	Defleshing	
				Anatidae		Humerus	1	Defleshing	
		XVIIc	MIS 9	Passeriformes		Ulna	2	Defleshing	
				Phasianidae		Humerus, ulna	2	Defleshing	
				Anas sp.	Duck	Coracoid, ulna	2	Defleshing	
	Gran Dolina	TD10.1	MIS 9	Aves, indet.		Long bone	1	Defleshing	[15]
				Passeriformes indet.		Tibiotarsus	1	Defleshing	
				Corvidae indet.		Humerus, tibiotarsus	2	Defleshing	
**Rest of Europe**									
	Lazaret	CII	190-150 ka	*Columba livia*	Rock dove	Humerus	1		[16]
	Krapina	9	~130 ka	*Haliaetus albicilla*	White-tailed eagle	Talons	8	Jewelry	[17]
						Phalanx	1		
	Pech de l'Azé IV	8	MIS 5 (100 ka)	-	Medium-size raptor	Phalanx	1	Non-subsistence type of activity	[18]
	Combe-Grenal	52	MIS 5b (c. 90 ka)	*Aquila chrysaetos*	Golden eagle	Talon	1	Symbolic behavior?	[19]
	Pié Lombard		MIS3-4 (c. 70 ka)	*Columba livia*	Rock dove	Several elements	23		[20]
				Pyrrhocorax sp.	Chough	Several elements	23		
				Alectoris graeca	Partridge	Several elements	6		
	Les Fieux	K (west area)	MIS3	*Aquila chrysaetos*	Golden eagle	Femur	1	Defleshing	[21]
		Ks	MIS3	*Aegypius monachus*	Cinereous vulture	Penultimate phalanx	1	Symbolic? (talon explotation?)	[22]
		Ks	MIS3	*Corvus corax*	Common raven	Tibiotarsus	1	Defleshing	
		I/J, Jbase, Ks, Recent cleaning	MIS3 (60-40 ka)	*Haliaeetus albicilla*	White-tailed eagle	Talon	5	Symbolic behavior?	

		I/J, Jbase, Ks, Recent cleaning	MIS3 (60-40 ka)	*Haliaeetus albicilla*	White-tailed eagle	First phalanx	1	Related to talon explotation? Symbolic behavior?	
	Grotte de l'Hyène		MIS3?	*Aquila chrysaetos*	Golden eagle	Talon	1		Mourer-Chauviré, personal communication (in[23])
	Grotte du Noisetier		MIS3	*Falco* sp.	Falcon	Humerus	1	Defleshing	[19]
	Salzgitter-Lebenstedt		MIS3 (c. 50 ka)	*Cygnus* sp.	Swan	Metacarpus	1		[24]
				Anas sp.	Dubbling duck	Humerus	1		
	Baume de Gigny	XV	50? ka	*Cygnus cygnus*	Whooper swan	Phalanx	1		[25]
	Pech de l'Azé I	4	46.8-44.5 ka cal BP	*Aquila chrysaetos*	*Golden eagle*	First phalanges	2		[26]
	Grotte du Renne, Arcy-sur-Cure	Xb	44-42 ka cal BP	*Cygnus cygnus*	Whooper swam	Ulna	1	-	Vanhaeren et al., forthcoming (in [27]) [28]
		IX-X	44-42 ka cal BP	*Haliaetus albicilla*	White-tailed eagle	Pedal phalanx	8	Talon extraction	
		IX-X	44-42 ka cal BP	*Bubo bubo*	European eagle-owl	Pedal phalanx	1	Talon extraction	
		IX	44-42 ka cal BP	*Gyps fulvus*	Griffon vulture	Radius	1	-	
		Xa	44-42 ka cal BP	*Gyps fulvus*	Griffon vulture	Radius	1	-	
		Xb	44-42 ka cal BP	*Gyps fulvus*	Griffon vulture	Humerus	2	-	
		Xc	44-42 ka cal BP	*Gyps fulvus*	Griffon vulture	Metaphysis	1	-	
		Xc	44-42 ka cal BP	Indet.	Eagle or swan	Radius	1	-	
		VIII	44-42 ka cal BP	*Gyps fulvus*	Griffon vulture	Ulna	1	-	
		VIII	44-42 ka cal BP	Indet.	N/A	Diaphysis	1	-	
		IXb	44-42 ka cal BP	Indet.	Indet.	Diaphysis	n/a	-	
		IXb	44-42 ka cal BP	Indet.	Small bird	Diaphysis	1	-	
	Mandrin	E	MIS3 (c. 55-46 ka)	*Aquila chrysaetos*	Golden eagle	Talon		Symbolic expression	[29]
	Grotta del Rio Secco	7	MIS3 (c. 49.1-48 ka)	*Aquila chrysaetos*	Golden eagle	Talon	1	Symbolic expression	[29]
	Fumane	A4-A3	44.8-42.2 ka cal BP	*Aquila chrysaetos*	Golden eagle	Ulna	1	Symbolic (feathers)	[30]
		A6-A5	44.8-42.2 ka cal BP	*Gypaetus barbatus*	Bearded vulture	Ulna	1	Symbolic (feathers)	
				Falco vespertinus	Red-footed falcon	Humerus	1	Symbolic (feathers)	

				Columba palumbus	Common wood pigeon	Carpometacarpus	1	Symbolic (feathers)	
				Pyrrhocorax graculus	Alpine chough	Ulna	2	Symbolic (feathers)	
		A9*	47.6-45 ka cal BP	cf. *Aegypius monachus*	Cinereous vulture	Humerus and carpometacarpus	1	Utilization of bones	[31]
				cf. *Gypaetus barbatus*	Bearded vulture	Humerus	1	Recovery of feathers and/or defleshing	
				Aquila clanga	Greater spotted eagle	Radius	1	Recovery of the bone/wing	
				Tetrao tetrix	Black grouse	Several elements	4	Defleshing, feathers, talons, etc.	
				Pica pica	Common magpie	Tibiotarsus	1	Recovery of tendons?	
				Pyrrhocorax graculus	Chough	Several elements	10	Defleshing	
		Base of level A	MIS3	*Aquila chrysaetos*	Golden eagle	Talon	1	Ornament (talon)	
	Zaskalnaya VI	III	MIS 3 (c. 38-43 ka)	*Corvus corax*	Common raven	Radius	1	Symbolic function	[27]

*Fossil remains with probable cut-marks are not included.
In Payre, levels Ga/Gb (MIS8-7) feather barbules were found in the lithic residue[32]. In level 4 of Abri du Maras, feather barbules belonging to Accipitriformes and Anseriformes were found in the lithic residue[33].
[a]The data include here cf. *Columba livia/oenas* and *Columba palumbus*.
[b]Including *Pyrrhocorax graculus* and *Pyrrhocorax pyrrhocorax*.
[c]*Corvus monedula, Corvus corone/frugileus*, *Corvus corax* and *Pica pica*.
[d]Including both *Pyrrhocorax pyrrhocorax* and *P. graculus*; and *Corvus monedula* and *Corvus* sp.

Table S8

Selected European Middle Paleolithic, Jerzmanowician and Chatelperronian sites[a] with cut-marks on terrestrial carnivore remains.

Site	Level/Unit	Chronology	Taxon	Common name	Element(s)	NISP with cut-marks	Interpretation	Reference
**Iberian Peninsula**								
Axlor	III	MIS 3	cf. *Lynx* sp.	Lynx	Femur	1	Defleshing	This study
	V	MIS 3	cf. *Canis lupus*	Wolf	Radius	1	Defleshing/skinning	This study
Gran Dolina	TD10.1	MIS 9	*Panthera leo fossilis*	Lion	Rib, phalanx	2	Skinning and defleshing	[34]
			Vulpes vulpes	Fox	Coxal bone	1	Dismembering	[15]
			Canis/Cuon	Wolf/Dhole	Femur	1	Defleshing	[35]
Valdegoba		MIS 3-5	Canidae indet.	Indeterminate canid		2		[7]
			Carnivora indet.	Indeterminate carnivore		1		
Maltravieso	Sala de los Huesos	183-177 ka BP	*Crocuta crocuta*	Spotted hyena	Several elements	3	Butchering	[36,37]
Cova Negra		Middle Pleistocene	*Panthera pardus*	Leopard	5th metatarsal	1	Skinning	[38]
			Cuon cf. *alpinus*	Dhole	Mandible	1		[5]
Bolomor	IV	>120 ka BP	*Vulpes vulpes*	Fox	Fibula	1	Defleshing	[15]
			Panthera leo spelaea	Cave lion	Fibula	2	Defleshing	
			Lynx pardinus	Iberian lynx	Phalanx	1	Skinning	
			Carnivora indet		Fibula	1	Defleshing	
Abric Romaní	O	MIS 3 (c. 55 ka)	*Felis silvestris*	Wildcat	Tibia, mandible, two second phalanges	4	Skinning, defleshing.	[39]
Zarzamora		>44 ka BP	*Lynx* sp.	Lynx	Humerus	1	-	[40]
**Rest of Europe**								
Biache-Saint-Vaast		MIS 7	*Ursus deningeri*	Deninger's bear	Several elements	2496	Defleshing	[41]
			Ursus arctos	Brown bear	Several elements			
Lazaret		MIS 6	*Lynx spelaea*	Lynx	Femur, mandible	2	Filleting, skinning	[42]

	Grotte des Cèdres		MIS6?	*Ursus thibetanus*	Asian black bear		3	Skinning/Defleshing	Bez, 1995 (in [43]) [44]
				Panthera spelaea	Cave lion	Second phalanx	1	Skinning	
	Taubach	Bone sand	MIS 5e or older	*Ursus arctos*	Brown bear	Several elements	292	Defleshing, skinning	[45]
	Krapina		~130 ka	*Ursus spelaeus* + *U. arctos*	Cave and brown bears	Several elements	25	Defleshing, skinning, dismembering	[46]
				Canis lupus	Wolf	Radius	1	Disarticulation/skinning	
	Scladina	5	MIS5d-5b	*Ursus spelaeus*	Cave bear	Femur, tibia	6	Defleshing. Retouchers	[47]
	Caverna delle Fate		MIS 5-4?	*Ursus spelaeus*	Cave bear	Several bones	12	Skinning, defleshing, dismembering. Retouchers	[44]
	Regourdou	IV	MIS 5	*Ursus arctos*	Brown bear	Tibia, ribs	3	Skinning/Defleshing	[43,48]
	Madonna del l'Arma		MIS 5	*Ursus spelaeus*	Cave bear	Mandible	1	Skinning	[42]
	Les Pradelles	10 (facies 2a in recent excavations)	MIS 4? (72-60 ka BP)	*Crocuta crocuta*	Spotted hyena	Femur	1	Possible example of numerical notation	[49]
	Arma delle Manie		MIS 4?	*Ursus arctos*	Brown bear	Fibula	1	Filleting	[42]
				Ursus sp.	Bear		2		
	Grotta di Badalucco		Middle Paleolithic	*Ursus spelaeus*	Cave bear	Rib	1		[50]
	Grotta del Rio Secco	5 top + 7	49-42 ka cal BP	*Ursus spelaeus* + *U. arctos*	Cave and brown bears	Several elements	29	Defleshing, skinning	[51]
		5 + 8	49-42 ka cal BP	*Ursus spelaeus* + *U. arctos*	Cave and brown bears	Several elements	23	Defleshing, skinning	
	Fumane	A5-A5+A6	44.8-42.2 ka cal BP	*Ursus spelaeus* + *U. arctos*	Cave and brown bears	Several elements	6	Defleshing, skinning, retouchers	[51]
		A6	44.8-42.2 ka cal BP	*Ursus spelaeus* + *U. arctos*	Cave and brown bears	Several elements	8	Defleshing, skinning, retouchers	
		A5-A5+A6	44.8-42.2 ka cal BP	*Vulpes vulpes*	Red fox	Several elements	4	Butchering	[52]
		A5-A5+A6	44.8-42.2 ka cal BP	cf. *Vulpes lagopus*	Arctic fox	Hemimandible	1	Hide removal and muscle detachment	
	Pešturina	4	44.6-43.5 ka cal BP (^{14}C)/ 102.5-93.5 BP (ESR	*Ursus spelaeus*	Cave bear	Second cervical vertebra (axis)	1		[53]
	Salzofen		Middle Paleolithic	*Ursus spelaeus*	Cave bear	Femur	1		[54]

	Divje babe I	Several levels	Mousterian	*Ursus spelaeus*	Cave bear	Several elements	15		[55]
	Geissenklösterle		43 ka BP or more	*Canis lupus*	Wolf		1		[56]
				Ursus arctos	Brown bear		1		
				Ursus spelaeus	Cave bear		6		
	Hohle Fels		Middle Paleolithic	*Canis lupus*	Wolf		1		[56]
				Ursus spelaeus	Cave bear		5		
	Bockstein		Middle Paleolithic	*Crocuta crocuta*	Cave hyena		1		[56]
				Ursus spelaeus	Cave bear		2		
	Grotta di Moscerini	M5	Middle Paleolithic	*Ursus arctos*	Brown bear	Second phalanx	1		[57]
	Grotta di Sant Agostino	S1	Late Middle Paleolithic	*Ursus arctos*	Brown bear	First phalanx	1		[57]
	Goyet		Middle Paleolithic	*Ursus spelaeus*	Cave bear	Pisiform, metacarpal	2	Skinning	[58]
	Le Portel-Ouest		Mousterian[c]	*Ursus spelaeus*	Cave bear		1		[59]
			Mousterian[c]	*Canis lupus*	Wolf		1		
		F2	Mousterian	*Panthera spelaea*	Lion	Radius	1		
	Chez-Pinaud Jonzac	22	Quina Mousterian	*Vulpes* sp.	Fox	Tibia	1	Disarticulation, skinning	[60]
				Panthera spelaea	Lion	Metatarsal	1		Beauval, 2004 (in [60])
	Nietoperzowa		Mousterian	*Ursus spelaeus* s.l.	Cave bear s.l.	Metapodials, radius	-	Skinning and dismembering	Wojtal, 2007 (in [61])
	Font-de-Gaume III	4-5	Chatelperronian	*Ursus spelaeus*	Cave bear	Several elements	15 (NMI=3)		[62]
	Grotte du Renne, Arcy-sur-Cure	IX & X	Chatelperronian	*Ursus spelaeus*	Cave bear				[63]
	Mamutowa		Jerzmanowician	*Ursus spelaeus* s.l.	Cave bear s.l.	Metapodials (cut-marks), Radius (chopping marks)	-	Skinning and dismembering	Wojtal, 2007 (in [61])

[a]Or of attributable chronology.

[b]It is not possible to currently completely associate this level to the Mousterian.

[c]Gardeisen[59] does not provide the level from which these remains derive. The sequence at le Portal-Ouest has yielded several layers of Mousterian occupations and one layer is attributed to the Chatelperronian.

References

1 Mozota Holgueras, M. El utillaje óseo musteriense del nivel “D” de Axlor (Dima, Vizcaya): análisis de la cadena operativa. *Trabajos de Prehistoria* **66**, 28–46 (2009).

2 Castaños, P. M. in *Neandertales cantábricos, estado de la cuestión* (eds J.A. Lasheras & R. Montes) 201–207 (Museo de Altamira, 2005).

3 Barandiarán, J. M. in *Obras Completas de José Miguel de Barandiarán Tomo XVII* (ed J.M. Barandiarán) 127–384 (La Gran Enciclopedia Vasca, 1980).

4 Altuna, J. in *L'Homme de Neandertal. La Subsistance. Actes du Colloque International de Liège, vol. 6* (eds M. Pathou & L.G. Freeman) 41–43 (ERAUL, 1989).

5 Pérez Ripoll, M., Morales Pérez, J. V., Sanchis Serra, A., Aura Tortosa, J. E. & Sarrión Montañana, I. Presence of genus *Cuon* in upper Pleistocene and initial Holocene sites of the Iberian Peninsula: new remains identified in archaeological contexts of the Mediterranean region. *J. Archaeol. Sci.* **37**, 437–450 (2010).

6 Altuna, J. Hallazgo de un cuon (*Cuon alpinus* Pallas) en Obarreta, Gorbea (Vizcaya). *Kobie* **XIII**, 141–158 (1983).

7 Díez, C. Huellas de descarnado en el Paleolítico Medio: la cueva de Valdegoba (Burgos). *Zona Arqueológica* **7**, 304–317 (2006).

8 Finlayson, C. *et al.* Birds of a feather: Neanderthal exploitation of raptors and corvids. *PLoS ONE* **7**, e45927 (2012).

9 Blasco, R. *et al.* The earliest pigeon fanciers. *Sci. Rep.* **4**, 5971 (2014).

10 Blasco, R., Rosell, J., Rufà, A., Sánchez Marco, A. & Finlayson, C. Pigeons and choughs, a usual resource for the Neanderthals in Gibraltar. *Quat. Int.* **421**, 62–77 (2016).

11 Martínez Valle, R., Guillem Calatayud, P. M. & Villaverde Bonilla, V. Bird consumption in the final stage of Cova Negra (Xátiva, Valencia). *Quat. Int.* **421**, 85–102 (2016).

12 Blasco, R. & Fernández Peris, J. Small and large game: Human use of diverse faunal resources at Level IV of Bolomor Cave (Valencia, Spain). *Comptes R. Palevol.* **11**, 265–282 (2012).

13 Blasco, R. & Fernández Peris, J. Middle Pleistocene bird consumption at Level XI of Bolomor Cave (Valencia, Spain). *J. Archaeol. Sci.* **36**, 2213–2223 (2009).

14 Blasco, R. *Estrategias de Subsistencia de los Homínidos del Nivel XII de la Cova del Bolomor (La Valldigna, Valencia),* DEA thesis, Universitat Rovira i Virgili, (2006).

15 Blasco, R. *et al.* Environmental availability, behavioural diversity and diet: a zooarchaeological approach from the TD10-1 sublevel of Gran Dolina (Sierra de Atapuerca, Burgos, Spain) and Bolomor Cave (Valencia, Spain). *Quat. Sci. Rev.* **70**, 124–144 (2013).

16 Roger, T. *L'avifaune du Pléistocène moyen et supérieur du bord de la Méditerranée européenne: Orgnac 3, Lazaret (France), Caverne delle Fate, Arma delle Manie (Italie), Kalamakia (Grèce), Karain E (Turquie). Paléontologie, Taphonomie et Paléoécologie* Thèse de Doctorat thesis, (2004).

17 Radovčić, D., Sršen, A. O., Radovčić, J. & Frayer, D. W. Evidence for Neandertal jewelry: Modified white-tailed eagle claws at Krapina. *PLoS ONE* **10**, e0119802 (2015).

18 Dibble, H. L. *et al.* A preliminary report on Pech de l'Azé IV, layer 8 (Middle Paleolithic, France). *PaleoAnthropology* **2009**, 182–219 (2009).

19 Morin, E. & Laroulandie, V. Presumed symbolic use of diurnal raptors by Neanderthals. *PLoS ONE* **7**, e32856 (2012).

20 Romero, A. J., Díez, J. C. & Brugal, J. P. Aves de caza. Estudio tafonómico y zooarqueológico de los restos avianos de los niveles musterienses de Pié Lombard (Alpes-Maritimes, Francia). *Munibe (Antropologia Arkeologia)* **68**, 73–84 (2017).

21 Gerbe, M. *et al.* in *Transitions, ruptures et continuités en Préhistoire, Actes du XXVIIème Congrès Préhistorique de France, Bordeaux-les Eyzies 31 mai-5 juin 2010. Volume 2.*

Paléolithique et Mésolithique (eds J. Jaubert, N. Fourment, & P Depaepe) 257-279 (Société Préhistorique Française, 2014).
22 Laroulandie, V., Faivre, J.-P., Gerbe, M. & Mourre, V. Who brought the bird remains to the Middle Palaeolithic site of Les Fieux (Southwestern, France)? Direct evidence of a complex taphonomic story. *Quat. Int.* **421**, 116–133 (2016).
23 Fiore, I., Gala, M. & Tagliacozzo, A. Ecology and subsistence strategies in the eastern Italian Alps during the Middle Palaeolithic. *Int. J. Osteoarchaeol.* **14**, 273–286 (2004).
24 Gaudzinski-Windheuser, S. & Niven, L. in *The evolution of hominin diets: Integrating approaches to the study of Palaeolithic subsistence* (eds J.-J. Hublin & M.P. Richards) 99–111 (Springer Science + Business Media, 2009).
25 Mourer-Chauviré, C. *Les oiseaux du Pléistocene moyen et supérieur de France* Ph.D. dissertation thesis, Université Claude Bernard-Lyon 1, (1975).
26 Soressi, M. *et al.* in *Les sociétés Paléolithiques d'un grand Sud-Ouest : nouveaux gisements, nouvelles méthodes, nouveaux résultats. - Actes des journées décentralisées de la SPF des 24-25 novembre 2006 Mémoire XLVII de la Société préhistorique française* (ed J. G. Bordes I. Ortega J. Jaubert) 95–132. (Société Préhistorique française, 2008).
27 Majkić, A., Evans, S., Stepanchuk, V., Tsvelykh, A. & d'Errico, F. A decorated raven bone from the Zaskalnaya VI (Kolosovskaya) Neanderthal site, Crimea. *PLoS ONE* **12**, e0173435 (2017).
28 Julien, M., Vanhaeren, M. & d'Errico, F. in *The Third Man. The Prehistory of the Altai* 110-121 (Éditions de la Réunion des Musées Nationaux, 2017).
29 Romandini, M. *et al.* Convergent evidence of eagle talons used by Late Neanderthals in Europe: A further assessment on symbolism. *PLoS ONE* **9**, e101278 (2014).
30 Peresani, M., Fiore, I., Gala, M., Romandini, M. & Tagliacozzo, A. Late Neandertals and the intentional removal of feathers as evidenced from bird bone taphonomy at Fumane Cave 44 ky B.P., Italy. *PNAS* **108**, 3888–3893 (2011).
31 Fiore, I. *et al.* From feathers to food: Reconstructing the complete exploitation of avifaunal resources by Neanderthals at Fumane cave, unit A9. *Quat. Int.* **421**, 134–153 (2016).
32 Hardy, B. L. & Moncel, M.-H. Neanderthal use of fish, mammals, birds, starchy plants and wood 125-250,000 years ago. *PLos ONE* **6**, e23768 (2011).
33 Hardy, B. L. *et al.* Impossible Neanderthals? Making string, throwing projectiles and catching small game during Marine Isotope Stage 4 (Abri du Maras, France). *Quat. Sci. Rev.* **82**, 23–40 (2013).
34 Blasco, R., Rosell, J., Arsuaga, J. L., Bermúdez de Castro, J. M. & Carbonell, E. The hunted hunter: the capture of a lion (*Panthera leo fossilis*) at the Gran Dolina site, Sierra de Atapuerca, Spain. *J. Archaeol. Sci.* **37**, 2051–2060 (2010).
35 Rodríguez-Hidalgo, A., Saladié, P., Ollé, A. & Carbonell, E. Hominin subsistence and site function of TD10.1 bone bed level at Gran Dolina site (Atapuerca) during the late Acheulean. *J. Quat. Sci.* **30**, 679–701 (2015).
36 Rodríguez-Hidalgo, A. The scavenger or the scavenged? *J. Taphonomy* **8**, 75–76 (2010).
37 Rodríguez-Hidalgo, A., Saladié, P., Canals, A., Marín, J. & Carbonell, E. in *Proceedings of the Hominid-Carnivore interactions during the Pleistocene International Congress* 81 (Institut Català de Paleoecologia Humana i Evolució social (IPHES), 2011).
38 Sanchis, A. *et al.* Pleistocene leopards in the Iberian Peninsula: New evidence from palaeontological and archaeological contexts in the Mediterranean region. *Quat. Sci. Rev.* **124**, 175–208 (2015).
39 Gabucio, M. J., Cáceres, I., Rodríguez-Hidalgo, A., Rosell, J. & Saladié, P. A wildcat (*Felis silvestris*) butchered by Neanderthals in Level O of the Abric Romaní site (Capellades, Barcelona, Spain). *Quat. Int.* **326-327**, 307–318 (2014).

40 Sala, N., Algaba, M., Arsuaga, J. L., Aranburu, A. & Pantoja, A. A taphonomic study of the Búho and Zarzamora caves. Hyenas and humans in the Iberian Plateau (Segovia, Spain) during the Late Pleistocene. *J. Taphonomy* **10**, 477–497 (2012).
41 Auguste, P. Chasse et charognage au Paléolithique moyen: l'apport du gisement de Biache-Saint-Vaast (Pas-de-Calais). *Bulletin de la Société Préhistorique Française* **92**, 155–168 (1995).
42 Valensi, P. & Psathi, E. Faunal Exploitation during the Middle Palaeolithic in south-eastern France and north-western Italy. *Int. J. Osteoarchaeol.* **14**, 256–272 (2004).
43 Cavanhié, N. L'ours qui a vu l'homme? Étude archéozoologique et taphonomique du site palélithique moyen de Regourdou (Montignac, Dordogne, France). *Paleo* **21**, 39–64 (2009-2010).
44 Defleur, A. & Crégut-Bonnoure, E. La grotte des Cèdres, commune du Plan d'Aups (Var). *Bulletin de la Societé Préhistorique Française* **87**, 270–274 (1990).
45 Bratlund, B. Taubach revisited. *Jahrbuch des Römisch-Germanischen Zentralmuseums Mainz* **46**, 61–174 (1999).
46 Miracle, P. *The Krapina Neandertal site. Zooarchaeology, taphonomy, and catalog of the faunal remains*. (Hrvatski Prirodoslovni Muzej, 2007).
47 Abrams, G., Bello, S. M., Di Modica, K., Pirson, S. & Bonjean, D. When Neanderthals used cave bear (*Ursus spelaeus*) remains: Bone retouchers from unit 5 of Scladina Cave (Belgium). *Quat. Int.* **326-327**, 274–287 (2014).
48 Pelletier, M. *et al.* Rabbits in the grave! Consequences of bioturbation on the Neandertal "burial" at Regourdou (Montignac-sur-Vézère, Dordogne). *J. Hum. Evol.* **110**, 1–17 (2017).
49 d'Errico, F. *et al.* From number sense to number symbols. An archaeological perspective. *Philosophical Transactions of the Royal Society B* **373**, 20160518 (2018).
50 Quiles, J. Tanières d'ours des cavernes (Carnivora, Ursidae) du pourtour méditerranéen: étude taphonomique et paléobiologique de huit assemblages du Pléistocène supérieur. *Paleo* **16**, 171–192 (2004).
51 Romandini, M. *et al.* Bears and humans, a Neanderthal tale. Reconstructing uncommon behaviors from zooarchaeological evidence in southern Europe. *J. Archaeol. Sci.* **90**, 71–91 (2018).
52 Romandini, M. *et al*. Late neandertals and the exploitation of small mammals in northern Italy: fortuity, necessity or hunting variability? *Quaternaire* **29**, 61–67 (2018).
53 Majkić, A., d'Errico, F., Milošević, S., Mihailović, D. & Dimitrijević, V. Sequential incisions on a cave bear bone from the Middle Paleolithic of Pešturina Cave, Serbia. *Journal of Archaeological Method and Theory* **25**, 69–116 (2018).
54 Ehrenberg, K. Vom dermaligen Forschungsstand in der Höhle am Salzofen. *Quartär* **10**, 237-251 (1958).
55 Turk, I. in *Divje babe I. Part 2* (ed I. Turk) (Inštitut za arheologijo ZRC SAZU, 2014).
56 Kitagawa, K., Krönneck, P., Conard, N. & Münzel, S. C. Exploring cave use and exploitation among cave bears, carnivores and hominins in the Swabian Jura, Germany. *J. Taphonomy* **10**, 439–461 (2012).
57 Stiner, M. C. *Honor among thieves: A zooarchaeological study of Neandertal ecology*. (Princeton University Press, 1994).
58 Germonpré, M. & Sablin, M. V. The cave bear (*Ursus spelaeus*) from Goyet, Belgium. *Bullettin de l'Institut Royal des Sciences Naturelles de Belgique, Sciences de la Terre* **71**, 209–233 (2001).
59 Gardeisen, A. Middle Palaeolithic Subsistence in the West Cave of "Le Portel" (Pyrénées, France). *J. Archaeol. Sci.* **26**, 1145–1158 (1999).

60 Niven, L. *et al.* Neandertal mobility and large-game hunting: The exploitation of reindeer during the Quina Mousterian at Chez-Pinaud Jonzac (Charente-Maritime, France). *J. Hum. Evol.* **63**, 624–635 (2012).
61 Wojtal, P., Wilczyński, J., Nadachowski, A. & Münzel, S. C. Gravettian hunting and exploitation of bears in Central Europe. *Quat. Int.* **359-360**, 58–71 (2015).
62 Armand, D., Plassard, F. & Prat, F. L'ours de Font-de-Gaume III et le problème de l'exploitation de l'ours des cavernes. *Département de Rhône, Muséum, Lyon, Cahiers scientifiques, Hors série* **2**, 103–110 (2004).
63 Baryshnikov, G. & David, F. Les ours des cavernes à Arcy-sur-Cure (Yonne, France). *Ursus (speleardos) spelaeus* Rosenmüller et Heinroth, 1794. *Quaternaire* **11**, 65–79 (2000).
